# Analysis of Acoustic Surface Wave Focused Unidirectional Interdigital Transducers Using Coupling-of-Mode Theory

**DOI:** 10.3390/mi16010003

**Published:** 2024-12-24

**Authors:** Guopeng Hui, Tinglun Ao, Haotian Liu, Minglei Li, Chen Chen

**Affiliations:** School of Physics and Electronic Information, Yunnan Normal University, No. 1 Yuhua Area, Chenggong District, Kunming 650500, China; guopenghui920@126.com (G.H.); tinglun13356151578@163.com (T.A.); lht1277580916@163.com (H.L.); lml2096211@163.com (M.L.)

**Keywords:** coupling-of-mode theory, electrode width-control floating electrode unidirectional transducer, finite element method, electric potential standing wave, surface acoustic wave, transduction center, reflection center

## Abstract

In cell or droplet separation, high acoustic wave energy of a surface acoustic wave (SAW) device is required to generate sufficient acoustic radiation force. In this paper, the electrode width-control floating electrode focused unidirectional interdigital transducer (EWC-FEFUDT) is proposed due to its enhanced focusing properties. The performance of the EWC-FEFUDT is investigated using the Coupling-of-Mode (COM) theory, and the COM parameter is extracted using the Finite Element Method (FEM). The four different forbidden band edge frequencies account for the unidirectionality of the proposed EWC-FEFUDT. A direction angle of $\phi_{\kappa }-\phi_{\zeta }=44.5{}^{\circ}$ of the EWC-FEFUDT (Design 3) is obtained, being fairly close to the optimum value of 45°. The EWC-FEFUDT (Design 3) has a lower insertion loss (IL) of −5.1 dB and greater unidirectionality (20 × log_10_(D) = 13.8 dB). The SAW maximum amplitude of the EWC-FEFUDT (Design 3) is increased by about $1.5\times 10^{-4}\;µm$ compared to that of the focused interdigital transducers (FIDTs). The maximum acoustic pressure of the EWC-FEFUDT is an order of magnitude higher than that of FIDTs. The EWC-FEFUDT exhibits enhanced focusing properties. The proposed EWC-FEFUDT may provide an alternative method for cell or droplet separation in an efficient manner.

## 1. Introduction

**Figure 1:**
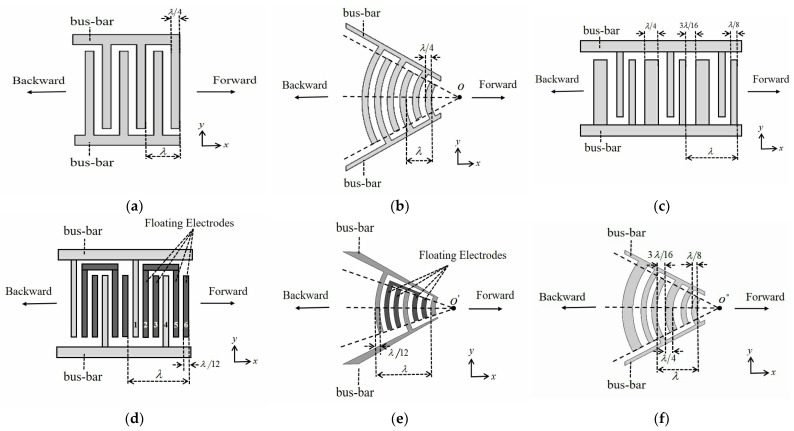
Various types of IDTs. (**a**) Conventional uniform IDT; (**b**) FIDT; (**c**) EWC-UDT; (**d**) FEUDT; (**e**) SPFT; (**f**) FUDT.

Recently, surface acoustic wave (SAW) devices have been widely used in passive wireless identification systems, acoustic sensors, and surface acoustic wave microfluidics [1,2,3,4,5,6,7,8,9,10]. Passive wireless identification systems require good directionality of the SAW. SAW sensing and microfluidic device performance is expected to have lower losses and better energy utilization efficiency. These put a high requirement on the design of the interdigital transducers (IDTs) of the SAW device. IDTs, which are important units in SAW devices for generating or receiving SAWs, can convert electrical energy to mechanical energy using the inverse piezoelectric effect or convert mechanical energy to electrical energy using the positive piezoelectric effect. Figure 1a shows the illustration of the conventional uniform IDT; each electrode of the IDT has a width of λ/4, where λ is the acoustic wavelength. These electrodes are periodically arranged and connected to the busbar. It produces a SAW with equal forward and backward amplitudes. Therefore, the triple-transit signal generated by the conventional IDT because of SAW reflection increases the insertion loss (IL) of the SAW device, reduces the IDT energy transfer efficiency, and destroys the directionality of SAW propagation. Figure 1b shows the focused interdigital transducer (FIDT), in which each electrode shape is an arc. It can focus the SAW on the center O of the arc electrodes. Therefore, the SAW energy propagating in the x-direction will be enhanced, so the FIDT has been widely applied in the microfluidic field [9,11,12]. However, the increase in x-direction SAW energy is accompanied by an increase in IL owing to SAW diffraction effects [13].

A unidirectional IDT (UDT) is a type of IDT that can excite the SAW predominantly toward forward propagating. Due to its inherent unidirectionality, the enhanced forward-propagating SAW not only suppresses the triple-transit signal but also effectively decrease the IL of the IDT [14,15] and the energy utilization efficiency.

Currently, the UDTs with internal reflective electrodes include types of electrode width control (EWC) UDTs [16], floating electrode unidirectional IDTs (FEUDT) [17], single-phase focused IDTs (SPFT) [18], and focused unidirectional IDTs (FUDT) [19]. Figure 1c shows the EWC-UDT. Each period consists of two narrow electrodes with a width of λ/8 and a wide electrode with a width of λ/4. It achieves unidirectional propagation of acoustic waves by the reflection of wide electrodes [20]. Figure 1d shows the FEUDT, where each electrode has a width of λ/12. The active electrodes 1 and 4 are connected to the busbars, with electrodes 2 and 5 behaving like the short-circuited gratings are mutually connected and electrodes 3 and 6 behaving like the open-circuited gratings are isolated. For the excitation of the SAW, the active electrodes and the mutually connected electrodes 2 and 5 play an important role. Electrodes 2 and 5 makes the transduction center shift away from the reflection center, and the amplitude of the forward-propagating SAW is not equal to that of the backward-propagating SAW. When the forward-propagating SAW and the reflected SAW by the internal floating electrodes 3 and 6 superpose constructively, the resulting wave propagates in a single direction [21,22]. This reduces the IL and the influence of triple-transit signals. The SPFT shown in Figure 1e is a combination of the structural characteristics of both an FIDT and FEUDT. The phase of the forward-propagating wave in the SPFT and that of the reflected wave are the same, which enhances the energy of the forward-propagating SAW [18], and then the forward-propagating SAW is focused at point $O^{\prime }$. Figure 1f shows the illustration of the FUDT. The FUDT has an advantage over the EWC-UDT in that it can focus the forward-propagating SAW energy at point $O^{\prime \prime }$.

The SPFT designed by Zhong et al. [18] has the advantages of smaller electrode resistance and higher operating frequency. However, there are six arc-shaped electrodes in each period of the SPFT. The typical SAW devices include a lot of electrodes (hundreds or even thousands of electrodes). It is difficult to manufacture actual SPFT-based SAW devices. The FUDT designed by Destgeer et al. [19] enhances the focus properties of the SAW, which results in high-energy SAWs at the focusing center position. This UDT solves the problem of low energy transfer efficiency of conventional IDTs and is used in particle separation. However, the input power of the FUDT is 94 mW, and the particle separation efficiency is only close to 30%, which remains to be improved. In addition, Destgeer et al. do not analyze the performances of the FUDT such as its IL, directionality, and eigenmodes, etc. Although Lu et al. [23] analyze the IL of the designed FUDT and find that the FUDT has a smaller IL, the analyses of the directionality and eigenmodes of the FUDT are not comprehensive enough. The understanding about the performance characteristics of the FUDT is still lacking.

**Figure 2:**
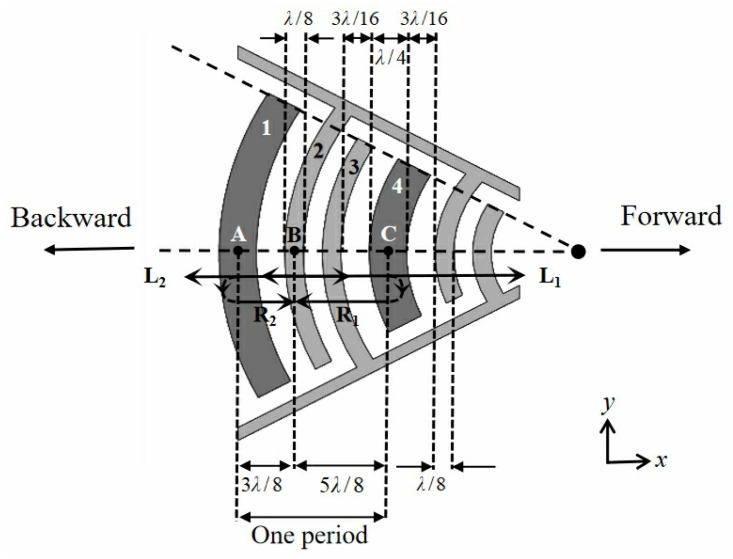
EWC-FEFUDT.

In order to improve the particle separation efficiency, high acoustic wave energy of a SAW device is required to generate sufficient acoustic radiation force acting on particles. The magnitude of acoustic wave energy depends on the structure of IDTs of the SAW device. Therefore, in this paper, we design the electrode width-control floating electrode focused unidirectional interdigital transducer (EWC-FEFUDT). As shown in Figure 2, each period of the EWC-FEFUDT consists of two narrow electrodes, 2 and 3, with a width of λ/8 and a wide floating electrode, 1, with a width of λ/4. All electrodes are arranged as a concentric circular arc. Compared with an SPFT, the EWC-FEFUDT is structurally simpler, and its wider floating electrode 1 can better enhance the reflection of the acoustic waves [19]. When a voltage is applied to electrode 2 and electrode 3 is grounded, the forward and backward SAWs (L_1_ and L_2_) are excited from the center (position B) of electrode 2. L_1_ and L_2_ are reflected as R_1_ and R_2_ when they respectively encounter the wide floating electrodes 4 and 1, of which the reflection points (A and C) are located in the centers. There is a phase difference between the reflected waves (R_1_ and R_2_) and the excited waves. The phase difference mainly consists of two parts: one is caused by the propagation distance, and the other is caused by electrode reflection [24]. Reflection includes mechanical reflection caused by the impedance discontinuity between the electrodes and the piezoelectric substrate and electrical reflection caused by the formation of a constant electric potential at the interface between the electrodes and the piezoelectric substrate. Electrical reflection is predominant and introduces a phase of π/2 during the propagation of SAWs. When R_1_ and R_2_ arrives at point B, they, respectively, propagate the distance of 5λ/4 and 3λ/4, and the corresponding phase differences are, respectively, 5π/2 and 3π/2. The reflected wave R_2_ and the excited wave L_1_ interfere constructively because their phase difference is 2π. The reflected wave R1 and the excited wave L_2_ interfere destructively because their phase difference is 3π [24]. The acoustic wave intensity of the forward-propagating SAW is greater than that of the backward-propagating SAW, which makes the EWC-FEFUDT unidirectional.

**Figure 3:**
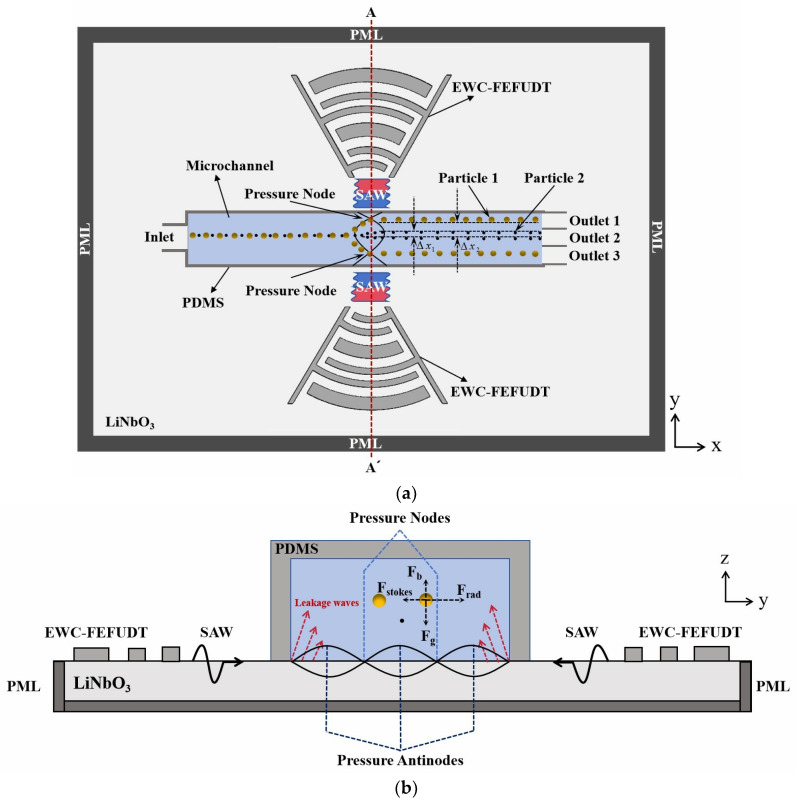
Schematic diagram of particle separation using EWC-FEFUDT (**a**) x–y plane view; (**b**) view of y–z cross-section taken along the line AA’ of (**a**).

The proposed EWC-FEFUDT provides an efficient method for particle separation. The schematic diagram of the particle separation device is shown in Figure 3. Two identical EWC-FEFUDTs are placed on each side of the microchannel. When they are stimulated with RF signals of equal magnitude but 180 degrees out of phase, two SAWs propagate in opposite directions along the surface of the piezoelectric substrate towards the fluid inside the microchannel. When the SAWs reach the fluid, leakage waves (as shown in Figure 3b) in a longitudinal mode are generated. The interference of the two SAWs forms the standing SAWs (SSAWs) that generate a periodic distribution of pressure nodes and antinodes. The particles in the fluid experience four forces, namely gravity $F_{g}=\rho_{p}Vg$ ($\rho_{p}$ is the particle density, and V is the volume of the particle), buoyancy $F_{b}=\rho_{f}Vg$ ($\rho_{f}$ is the fluid density), acoustic radiation force $F_{rad}=4\pi \phi \left(\tilde{\kappa },\tilde{\rho }\right)ka^{3}E_{ac}sin\left(2kx\right)$ ($\tilde{\kappa }$ is the relative compressibility, $\tilde{\rho }$ is the relative density, k is the acoustic wavenumber, $E_{ac}$ is the acoustic energy density, and x is the displacement of the particle) induced by the scattering of the acoustic waves, and stokes drag force $F_{stokes}=4\pi \eta a\left(v_{0}-v_{p}\right)$ (η is the fluid viscosity, a is the radius of the particle, and $v_{0}$ is the velocity of the fluid) arising from acoustic streaming. During the separation, the density of the particles is matched to the fluid density in order to avoid the sedimentation of particles, so the magnitudes of $F_{g}$ and $F_{b}$ are nearly the same. Because of force balance (in the vertical direction) of the particles, the displacement in the z-direction of the particles can be ignored, and only the lateral displacement is considered. When ideal fluid is considered, $F_{stokes}$ can be ignored, and then the movement of particles is dominated by the $F_{rad}$. The particles move to the pressure nodes or antinodes by the $F_{rad}$ depending on whether their acoustic contrast factor $\phi \left(\tilde{\kappa },\tilde{\rho }\right)=\frac{1}{3}\left[\left(5\tilde{\rho }-2\right)/\left(2\tilde{\rho }+1\right)-\tilde{\kappa }\right]$ ($\tilde{\kappa }=\kappa_{p}/\kappa_{f}$, $\tilde{\rho }=\rho_{p}/\rho_{f}$, where $\kappa_{p}$ and $\kappa_{f}$, respectively, represent the compressibility of the particle and fluid) is positive or negative, respectively [7]. Assume particles 1 (larger radius) and 2 with the same velocities of $v_{p}$ are introduced into the microchannel, and ϕ is positive. Then, they move to pressure nodes. Because larger particle 1 experiences a larger $F_{rad}$, so it has larger lateral displacement ($\Delta x_{1}$). Since $F_{rad}$ equals zero at the pressure node, the particles will no longer deflect and move at a constant velocity in the direction of the fluid flow. Due to their different lateral displacements ($\Delta x_{1}$ and $\Delta x_{2}$), the particles are separated successfully. Because higher acoustic wave energy is generated by the EWC-FEFUDTs, the particles will experience a larger $F_{rad}$. So, for constant lateral displacements, the particles move faster, and the separation efficiency is improved.

The eigenmodes, IL, focus properties, and directionality of the EWC-FEFUDT are investigated comprehensively. Firstly, the SAW propagation characteristics of the EWC-FEFUDT are analyzed theoretically using the Coupling-of-Mode (COM) theory, and then the parameters of the COM equations are determined using the Finite Element Method (FEM). The shift effects of the IDT and reflection centers due to the asymmetric electrode configurations are analyzed. The IL of the proposed EWC-FEFUDT is −5.1 dB, the orientation angle is 44.5°, and the directivity can reach 13.8 dB. The magnitude of the SAW amplitude in the focusing region of the EWC-FEFUDT is $1.5\times 10^{-4}\;µm$, which is much higher than that of the conventional FIDT, and the acoustic pressure of the interaction region of SAWs with fluid is an order of magnitude larger than that of the FIDT.

## 2. Methods

### 2.1. The Finite Element Method

**Figure 4:**
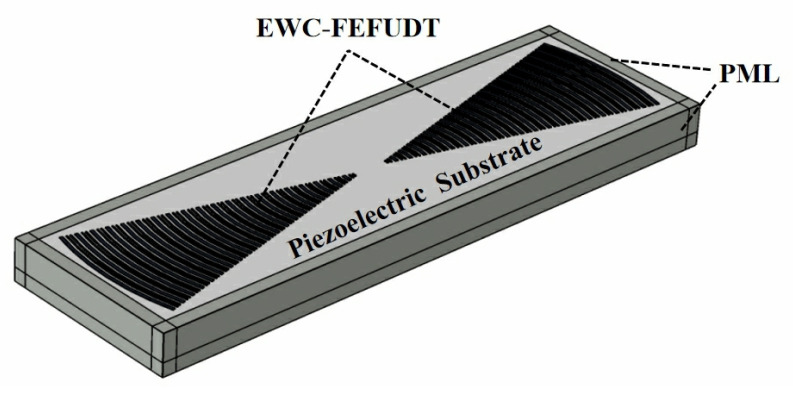
The complete FEM model of the EWC-FEFUDT.

The FEM is used to analyze the performance of the EWC-FEFUDT [25,26]. As shown in Figure 4, the complete FEM model of the EWC-FEFUDT consists of three parts: the EWC-FEFUDT with 24 pairs of electrodes, the piezoelectric substrate, and the perfectly matched layer (PML) for absorbing the SAWs [27] around the piezoelectric substrate.

The RF signal applied to the EWC-FEFUDT produces an electric field that generates SAWs on the surface of the piezoelectric substrate based on the inverse piezoelectric effect. In the piezoelectric substrate, the SAW generation is governed by the linear piezoelectric constitutive equations in stress-charge form, consisting of the momentum Equation (1) for mechanical vibration and the charge conservation Equation (2) for electrical behavior [28]:

(1) $$T_{ij}=c_{ijkl}^{E}S_{kl}-e_{ijk}^{T}E_{k}$$

(2) $$D_{i}=e_{ikl}S_{kl}+\varepsilon_{ik}^{S}E_{k}$$

where $T_{ij}$ represents the components of stress, $c_{ijkl}^{E}$ is the elastic constant for a constant electric field, $S_{kl}$ is the strain, $E_{k}$ is the electric field intensity, $D_{i}$ is electric displacement, $e_{ijk}^{T}$ is the piezoelectric constant, and $\varepsilon_{ik}^{S}$ is permittivity for constant strain. The subscripts i,j,k, and l represent four different directions in space, respectively. In the 3D space, the values of these subscripts are taken from the set {1, 2, 3}, where each number corresponds to a specific direction. The superscript T indicates that $e_{ijk}^{T}$ is the transpose of $e_{ijk}$. The superscripts E on $c_{ijkl}^{E}$ and S on $\varepsilon_{ik}^{S}$ indicate that they are the properties at a constant electric field and strain, respectively. The velocity of an acoustic wave is five orders of magnitude smaller than that of an electromagnetic wave. Therefore, the quasistatic approximation can be used to represent $E_{k}$ as:

(3) $$E_{k}=-\frac{\partial \phi }{\partial x_{k}}$$

where ϕ represents the electric potential.

When the piezoelectric substrate is not subjected to external forces, the equation of motion is given as [29]

(4) $$\frac{\partial T_{ij}}{\partial x_{j}}-\rho \frac{\partial^{2}u_{i}}{\partial t^{2}}=0$$

where ρ is the density of the piezoelectric substrate and $u_{i}$ represents the components of displacement.

The components of strain are defined by [29]

(5) $$S_{kl}=\frac{1}{2}\left(\frac{\partial u_{k}}{\partial x_{l}}+\frac{\partial u_{l}}{\partial x_{k}}\right)$$

By substituting Equations (3)–(5) into Equation (1), Equation (1) can be written as Equation (6):

(6) $$-\rho \frac{\partial^{2}u_{i}}{\partial t^{2}}+c_{ijkl}^{E}\frac{\partial^{2}u_{k}}{\partial x_{j}\partial x_{l}}+e_{ijk}^{T}\frac{\partial^{2}\phi }{\partial x_{k}\partial x_{j}}=0$$

In the case where there are no free charges in the piezoelectric substrate, the divergence of $D_{i}$ can be regarded as zero, as Equation (7) shows.

(7) $$\frac{\partial D_{i}}{\partial x_{i}}=0$$

By substituting Equations (3), (5), and (7) into Equation (2), Equation (2) can be written as Equation (8).

(8) $$e_{ikl}\frac{\partial^{2}u_{k}}{\partial x_{i}\partial x_{l}}-\varepsilon_{ik}^{S}\frac{\partial^{2}\phi }{\partial x_{i}\partial x_{k}}=0$$

**Figure 5:**
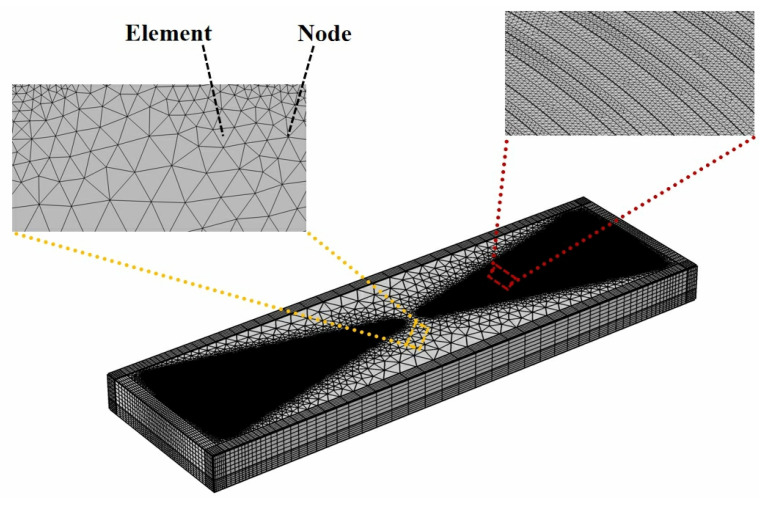
Elements and nodes of FEM model of EWC-FEFUDT.

Equations (6) and (8) can be discretized and solved to generate approximate solutions at each element or node of the FEM model of the EWC-FEFUDT, which has been divided into a finite number of elements, as shown in Figure 5.

**Figure 6:**
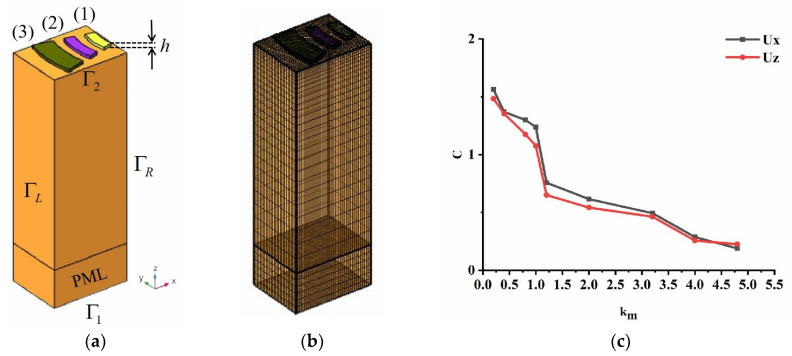
A single-period FEM model of the EWC-FEFUDT and the mesh convergence analysis. (**a**) A single-period FEM model of the EWC-FEFUDT; (**b**) meshes of a single-period FEM model of the EWC-FEFUDT; (**c**) mesh convergence analysis.

If all electrodes in the complete FEM model of the EWC-FEFUDT are considered in the calculation, the computational efficiency would be reduced because of a large number of elements. Therefore, the complete FEM model is simplified to a single-period model by using the periodic boundary conditions (PBCs) [30], as shown in Figure 6a, where h is the electrode thickness, and $\Gamma_{1},\;\Gamma_{2},\;\Gamma_{L},$ and $\Gamma_{R}$ are the boundaries of the model. Active electrodes 1 (yellow) and 2 (purple) are connected to the busbar, and 3 (brown) is the floating electrode. The width of the active electrode is fixed to λ/8, the width of the floating electrode is fixed to λ/4, and the wavelength λ is 80 µm. The normalized thickness of the electrodes h/λ is 1%, where h is the electrode thickness. The 128° Y-X LiNbO_3_ substrate has a length of λ, a width of 2λ/3, and a thickness of 6λ, and the thickness of the PML at the bottom is 2λ. The detailed electrical and mechanical boundary conditions for the model are given in Table 1.

To obtain more accurate solutions of the coupled wave equations, the meshes divided in the model must satisfy the convergence criterion. Therefore, the high-quality swept meshes are used for the piezoelectric substrate, and the tetrahedral meshes are used for the electrodes. The maximum mesh size of the substrate region is limited to $d/k_{m}$, where d is a predefined parameter, and $k_{m}$ is a custom parameter. In the calculation, d is set to one-sixth of the wavelength of the SAW. Figure 6b shows the structured meshes. It is important to investigate the mesh convergence because different mesh sizes result in different simulation results and times. The convergence parameter $C\left(F\right)$ is defined as [25]:

(9) $$C\left(F\right)=\sqrt{\frac{\int {\left(F-F_{ref}\right)}^{2}dxdz}{\int {F_{ref}}^{2}dxdz}}$$

where $F_{ref}$ is the reference solution obtained when $k_{m}=5$. The relationship between the convergence parameters $C\left(F\right)$ and $k_{m}$ is given in Figure 6c. $U_{x}$ and $U_{z}$ are the displacements in the x- and z-directions in the substrate, respectively. It can be seen that all variables begin to converge when $k_{m}$ reaches 1.2 and converge completely when the value of $k_{m}$ is taken from 4 to 5. In order to improve the computational efficiency, $k_{m}=4$ is used in this paper.

### 2.2. The Extraction of COM Equation Parameters Using the FEM

There are many methods for COM parameter determination, such as perturbation theory [13], wave theory [31], FEMSDA [31], etc. However, these methods are computationally intensive and cannot quickly extract the COM parameters of SAW devices. The FEM is versatile and easy to use because it can handle arbitrary materials and crystal cuts, different electrode shapes, and structures including multiple metal and dielectric layers [32,33,34]. In this paper, the COM parameters, including self-coupling coefficient ($\kappa_{11}$), mutual-coupling coefficients ($\kappa_{12}$), transduction coefficient (ζ), and static capacitance per unit length of the electrode (C) for the EWC-FEFUDT structure, are calculated based on the FEM.

From Equations (A48) and (A49) in Appendix A, $\left|\kappa_{12}\right|$ and $\kappa_{11}$ can be represented as:

(10) $$\left|\kappa_{12}\right|=\frac{2\pi }{\lambda }\frac{f_{s+}-f_{s-}}{f_{s+}+f_{s-}}$$

(11) $$\kappa_{11}=\frac{2\pi }{\lambda }\left(1-\frac{f_{s+}-f_{s-}}{2f_{0}}\right)$$

The velocity of SAWs can be calculated using the following equation:

(12) $$v=f_{0}\lambda$$

According to Equations (A50), (A55), and (A56) in Appendix A, the amplitude of the mutual-coupling coefficients in an open-circuited grating is written as:

(13) $$\left|\kappa_{b}\right|=\frac{2\pi }{\lambda }\frac{f_{o+}-f_{o-}}{f_{s+}+f_{s-}}$$

By substituting Equations (11) and (13) into Equation (A55) or (A56), the magnitude of a regeneration reflection term $2{\left|\zeta \right|}^{2}/\omega C$ can be obtained using the following equation:

(14) $$\frac{2{\left|\zeta \right|}^{2}}{\omega C}=\frac{2\pi }{\lambda }\left(\frac{f_{o+}+f_{o-}}{f_{s+}+f_{s-}}-1\right)$$

The expression for the reflection coefficient under the open-circuited condition can be written in a similar form to Equation (A42) in Appendix A.

(15) $$\kappa_{b}=\left|\kappa_{b}\right|\exp\left(j2\phi_{o}\right)$$

Substituting $\kappa_{b}=\kappa_{12}-2\zeta^{2}/\omega C$ and Equation (13) into Equation (15) gives the phase angle $\phi_{o}$ of the reflection coefficient under the open-circuited condition. Making full use of Equations (10), (13), and (14), $\phi_{o},$ and $\phi_{\kappa }$, we can get $\phi_{\zeta }$ [35]:

(16) $$\cos\left(2\phi_{\zeta }\right)=\frac{\left|\kappa_{b}\right|\cos\left(\phi_{o}\right)-\left|\kappa_{12}\right|\cos\left(\phi_{\kappa }\right)}{\frac{2{\left|\zeta \right|}^{2}}{\omega C}}$$

(17) $$\sin\left(2\phi_{\zeta }\right)=\frac{\left|\kappa_{b}\right|\sin\left(\phi_{o}\right)-\left|\kappa_{12}\right|\sin\left(\phi_{\kappa }\right)}{\frac{2{\left|\zeta \right|}^{2}}{\omega C}}$$

As can be seen from Equations (A42), (A43), (10), (11), (14), (16), and (17), if $f_{s-}$, $f_{s+}$, $f_{o-}$, and $f_{o+}$ are obtained, $\kappa_{11}$, $\kappa_{12}$, and ζ will be achieved. The $f_{s-}$, $f_{s+}$, $f_{o-}$, and $f_{o+}$ are calculated via the mode analysis of the single-period FEM model of the EWC-FEFUDT shown in Figure 6a, and the corresponding results are presented in Section 3.1.

When a voltage of 1 V is applied to the terminal electrode, the surface charge density $\sigma_{s}\left(x\right)$, the tangential component $E_{1}\left(x\right)$ of the electric field on the substrate surface, and the surface potential $\varphi \left(x\right)$ can be acquired [35] via the static analysis of the single-period EWC-FEFUDT model shown in Figure 6a. Then, the capacitance $C_{p}$ in the single-period EWC-FEFUDT structure can be calculated using:

(18) $$C_{p}=\frac{\int \sigma_{s}\left(x\right)dx}{\int E_{1}\left(x\right)dx}$$

The relationship between the static capacitance C per unit length of the electrode and $C_{p}$ is $C_{p}=C\lambda$. Therefore, all the parameters in the COM equation can be solved.

## 3. Results

### 3.1. Mode Analysis of EWC-FEFUDT

**Figure 7:**
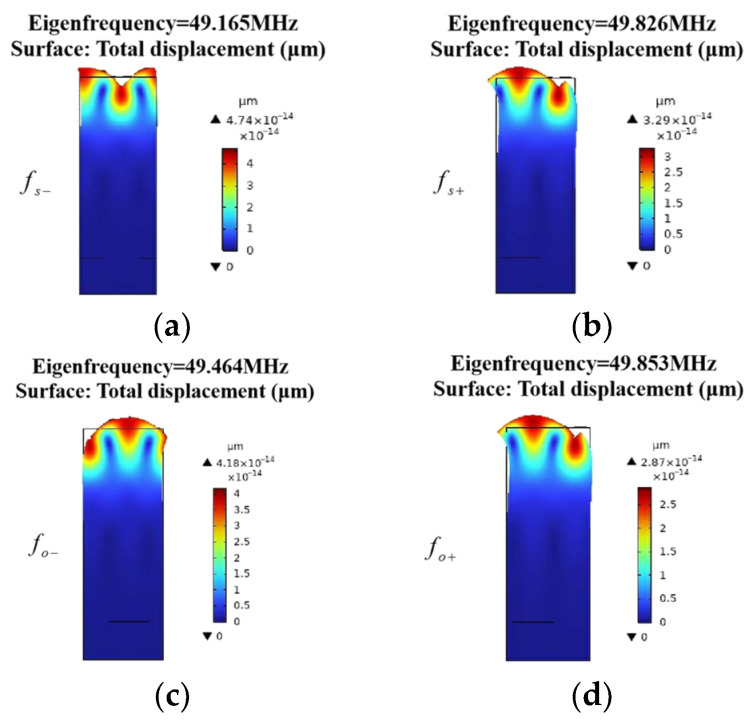
The total displacements of eigenmodes. (**a**) The lower frequency of the forbidden band in a short-circuited grating; (**b**) the upper frequency of the forbidden band in a short-circuited grating; (**c**) the lower frequency of the forbidden band in an open-circuited grating; (**d**) the upper frequency of the forbidden band in an open-circuited grating.

**Figure 8:**
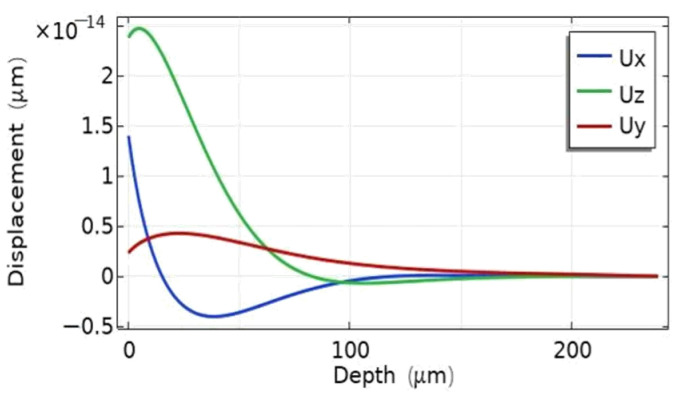
The displacement components of Figure 7a.

The mode analysis of the EWC-FEFUDT is conducted using the single-period EWC-FEFUDT model shown in Figure 6a. Figure 7 shows the total displacements of eigenmodes at the characteristic frequencies under short-circuit gratings and open-circuit gratings. The total displacement decreases to zero with a substrate thickness of about 2λ. Figure 8 shows the displacement components in Figure 7a. As can be seen, both $U_{x}$ and $U_{z}$ attenuate to zero within 2 to 3 wavelengths, and they are 90° out of phase with each other. The similar distributions of the displacement components in Figure 7b–d, which are omitted here, can be observed. These illustrate the important properties of the Rayleigh mode of SAWs. Note that although $U_{y}$ exists, it is significantly smaller compared with $U_{x}$ and $U_{z}$, so the mode can be approximately considered as the Rayleigh mode.

### 3.2. Frequency Response Analysis of the Admittance of the EWC-FEFUDT

The electrical performance of the SAW device can be characterized by the frequency response curve of the admittance, and the harmonic admittance can be determined from the calculation of the complete charge distribution on the EWC-FEFUDT, denoted by the admittance Y [36]:

(19) $$Y=j\frac{\omega Q}{V}$$

where Q is the electrical charge, and ω is the angular frequency.

**Figure 9:**
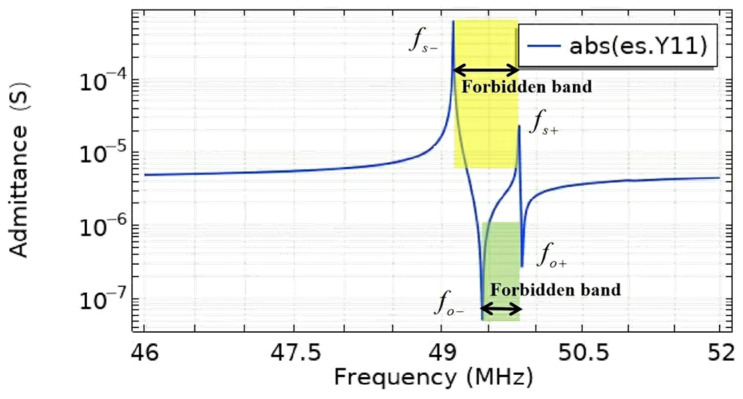
Admittance curves of EWC-FEFUDT on 128°Y-X LiNbO_3_.

The admittance frequency response curves of the EWC-FEFUDT are shown in Figure 9. The yellow region shows the forbidden bandwidth under short-circuit gratings. The green region shows the forbidden bandwidth under open-circuit gratings. The resonant frequencies $f_{s+}$ and $f_{s-}$ are 49.826 MHz and 49.165 MHz. The anti-resonance frequencies $f_{o+}$ and $f_{o-}$ are 49.853 MHz and 49.464 MHz. The values of $f_{s+}$, $f_{o+}$, $f_{s-}$, and $f_{o-}$ are exactly the same as those in Section 3.1. The four different forbidden band edge frequencies account for the unidirectionality of the EWC-FEFUDT [21].

### 3.3. Insertion Loss Analysis of EWC-FEFUDT

**Figure 10:**
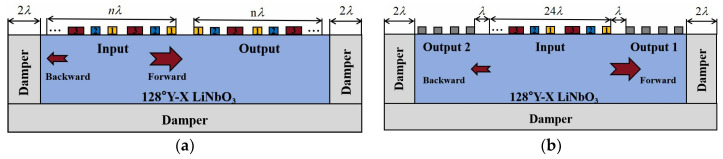
Two-dimensional FEM models of the SAW device. (**a**) The model for the analysis of the EWC-FEFUDT; (**b**) the model for the IL calculation of the EWC-FEFUDT.

IL is an important parameter for evaluating IDTs. The inherent IL of 3 dB in conventional bidirectional IDTs and the presence of triple-transit signals result in SAW devices with higher losses. However, a unidirectional IDT can reduce the losses generated by the triple-transit signals so that acoustic energy is efficiently utilized. The structural parameters of the IDT, such as the electrode pairs (n), the focused angle ($D_{a}$) and focused distance ($f_{L}$) of the IDT, the thickness of the electrodes (h), and the delay distance between the IDT (L), have significant influence on the IL. So, the ILs of the eleven different EWC-FEFUDT structures designed are investigated using the 3D FEM model shown in Figure 4. Figure 10a is the corresponding 2D cross-section of the 3D model. The detailed structural parameters of eleven different EWC-FEFUDTs are given in Table 2. The IL can be expressed as [37]:

(20) $$IL(dB)=20\times \log_{10}(\left|V_{out}/V_{in}\right|)$$

where $V_{out}$ is the output voltage, and $V_{in}$ is the input voltage.

**Figure 11:**
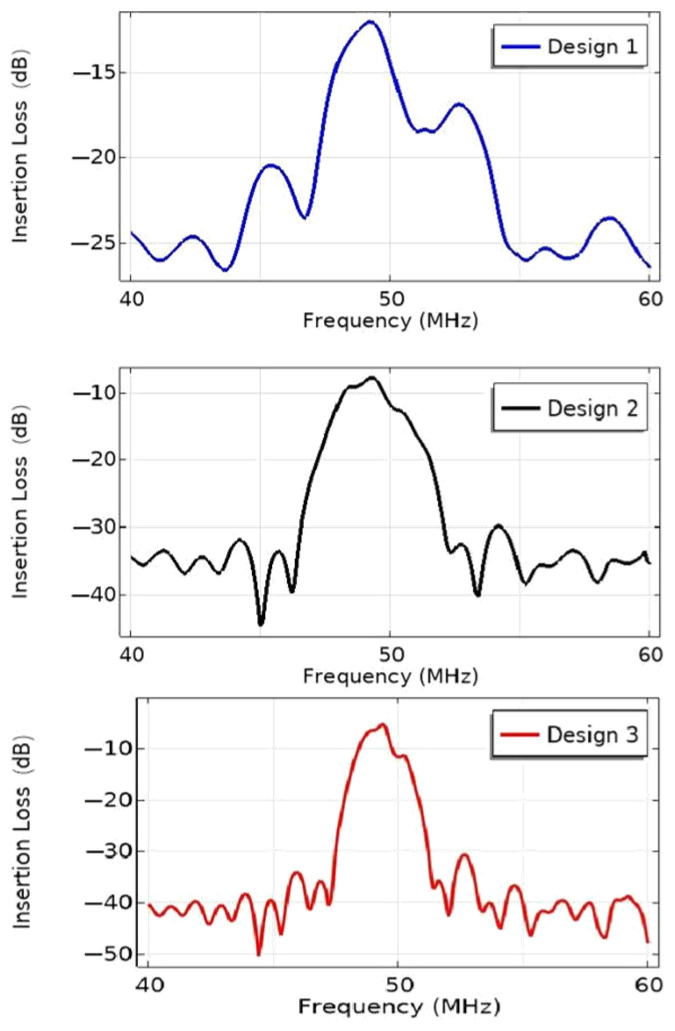
The effect of n on the IL of the EWC-FEFUDT.

The effect of the number of electrode pairs on the IL of the EWC-FEFUDT is shown in Figure 11. As shown in the figure, the increase in the number of electrode pairs leads to a narrowing of the bandwidth of the EWC-FEFUDT. The IL decreases with the increase in n. Since the acoustic waves excited by each electrode interfere with each other constructively, the greater the number of electrode pairs of the EWC-FEFUDT are, the stronger the produced acoustic waves are and the smaller the IL is.

**Figure 12:**
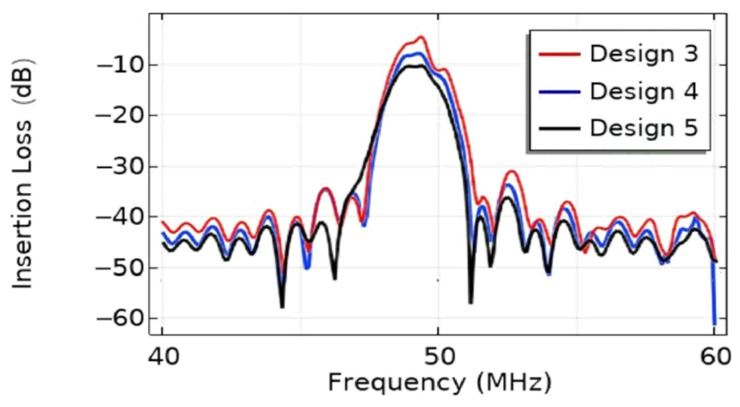
The effect of $D_{a}$ on IL of the EWC-FEFUDT.

The acoustic aperture of the EWC-FEFUDT is the width of acoustic radiation corresponding to the electrode with the shortest arc in the EWC-FEFUDT, and it is determined by $D_{a}$ and $f_{L}$. When $f_{L}=45\;µm$, the effect of $D_{a}$ on the IL is shown in Figure 12. As can be seen, the IL increases with the increase in $D_{a}$, but the bandwidth does not change significantly. An increase in $D_{a}$ results in an increase in SAW diffraction, which results from the high anisotropy of the piezoelectric substrate [38]. To obtain a lower loss, the $D_{a}$ should be smaller. Considering that the EWC-FEFUDT will be subsequently used in microfluidic chips for particle separation, the magnitude of the amplitude of the SAW generated by the EWC-FEFUDT in the focusing region is also one of the important parameters to be investigated, which will be discussed in Section 3.4.

**Figure 13:**
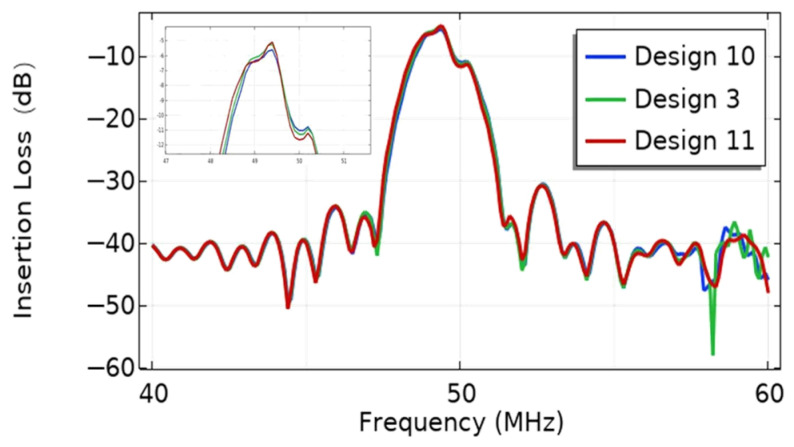
IL of EWC-FEFUDT with different electrode thicknesses.

Figure 13 demonstrates the effect of different h values on the IL of the EWC-FEFUDT. As can be seen, the IL is nearly the same when h=0.8 µm and 1.0 µm. In contrast, the IL is largest when h=0.6 µm, which is because the thinner metal electrode results in smaller mechanical reflections per unit period of the EWC-FEFUDT. Due to the mass loading effect, the h of the EWC-FEFUDT should not exceed 1 μm.

**Figure 14:**
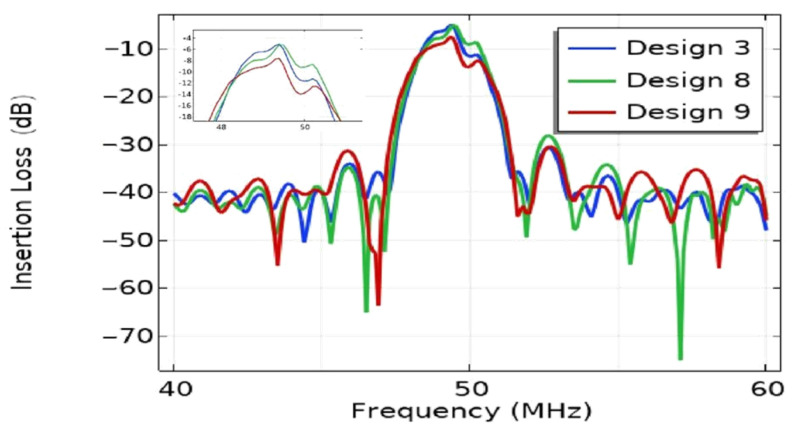
Effect of $f_{L}$ on IL of EWC-FEFUDT.

The effect of $f_{L}$ on the IL is shown in Figure 14. As expected, the EWC-FEFUD device with shorter $f_{L}$ shows decreased IL. The IL of Design 3 is, respectively, 0.2 dB and 2.7 dB lower than that of Design 8 and Design 9. It indicates that if $f_{L}$ continues to increase, the IL will also increase because of higher propagation losses, which are attributed to second-order effects arising from substrate anisotropy such as SAW diffraction, reflection, and beam steering, which are stronger in the EWC-FEFUDT in comparison to the conventional IDT [38].

**Figure 15:**
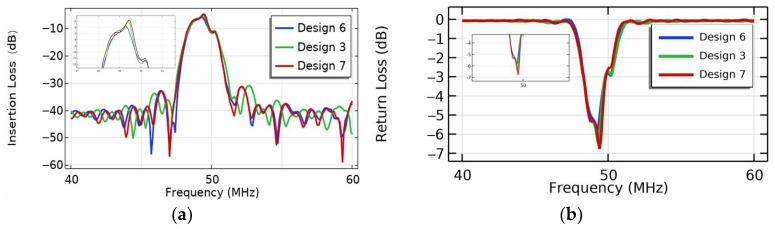
EWC-FEFUDT loss for different L. (**a**) Insertion loss; (**b**) return loss.

Figure 15 exhibits IL of the EWC-FEFUDT for different L values. Normally, the longer L will result in the larger propagation loss, so the IL will also increase. However, in the Figure 15a, the IL of Design 6 is significantly larger than that of Designs 7 and 3; that is, the IL does not increase with an increasing L. This can be explained as follows: Due to the impedance mismatch between the substrate material and the electrode material of the SAW device, part of the SAW propagating to the receiving UDT is reflected by the electrode [39]. The magnitude of the reflected wave can be expressed in terms of the return loss (RL).

(21) $$RL\left(dB\right)=20\times \log_{10}\left[\left(V_{in}-\left|V_{out}\right|\right)/V_{in}\right]$$

According to the energy conservation law, the greater the IL of the EWC-FEFUDT, the greater the $V_{out}$ in Equation (21), the smaller the RL [40]. In Figure 15b, the RL values of Design 6 are significantly smaller than those of Designs 7 and 3; that is, the reflected SAW energy in Design 6 is the greatest. Therefore, the more SAW energy reflected by the receiving electrode, the smaller the RL and the greater the IL of the EWC-FEFUDT, namely.

**Figure 16:**
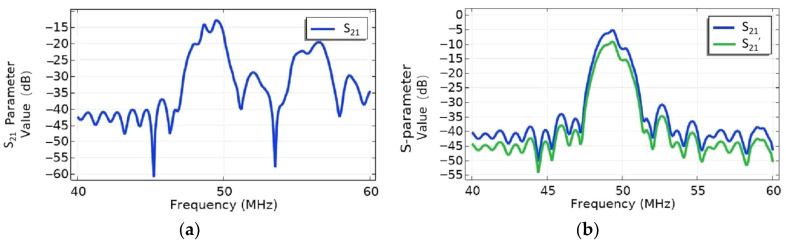
S parameters. (**a**) FIDT; (**b**) EWC-FEFUDT.

Figure 16 gives the comparison of the S parameters between the EWC-FEFUDT (Design 3) and FIDT with the same structural parameters. The calculation model is shown in Figure 10b, in which the output IDTs are placed on both sides to receive the SAWs. The S_21_ parameter represents the forward IL for both the EWC-FEFUDT and FIDT; the S_21_′ parameter represents the backward IL of the EWC-FEFUDT. Comparing Figure 16a,b, the IL value of the FIDT is 7 dB higher than that of the EWC-FEFUDT at the resonance frequency. The difference between the S_21_ and S_21_′ at the center frequency of the EWC-FEFUDT in Figure 16b is approximately 4 dB. The unequal IL in both directions also indicates the unidirectional of the EWC-FEFUDT.

### 3.4. Analysis of Focus Properties of EWC-FEFUDT

**Figure 17:**
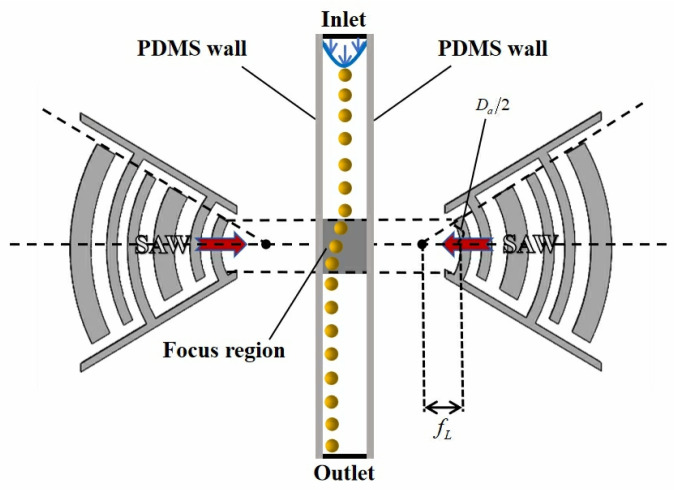
Focus region of EWC-FEFUDT.

**Figure 18:**
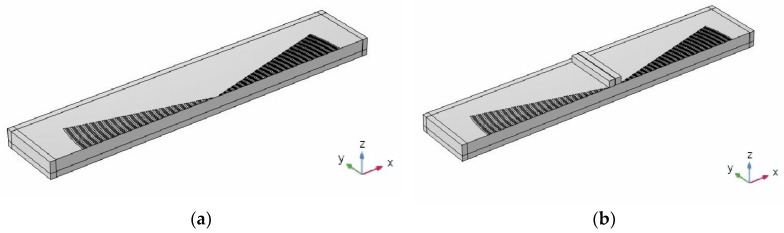
The half-symmetry FEM model of the EWC-FEFUDT. (**a**) The model for the analysis of SAW displacement; (**b**) the model for the analysis of acoustic pressure.

The EWC-FEFUDT can produce higher forward energy transmission than the conventional IDT. The reason is that the special circular arc structure of the EWC-FEFUDT allows for the better concentration of SAW energy in the focus region. The focus region in the microchannel is defined as the shaded region in Figure 17. Two SAWs propagating towards each other interfere in this region, thereby forming SSAWs. When particles pass through this region, they will be affected by the acoustic radiation force generate by the SSAW field and produce lateral displacement. The size of the focal region of the EWC-FEFUDT is determined by $f_{L}$ and $D_{a}$. To improve the computational efficiency, the half-symmetry FEM model, by imposing the symmetric boundary conditions shown in Figure 18, is used to analyze the focus property of the EWC-FEFUDT. The model in Figure 18a is used to analyze the amplitude magnitude of the SAW in the substrate. In Figure 18b the rectangular microfluidic channel made of polydimethylsiloxane (PDMS) is added for analyzing the acoustic pressure field. It has a width of 200 μm in the x-direction, a length of 2340 μm in the y-direction, and a height of 100 μm in the z-direction. When the SAW propagates to the PDMS walls, part of the SAW is reflected due to the impedance mismatch between PDMS and the piezoelectric substrate, and the resonance peak in the focal region will decrease. Furthermore, for a fixed wavelength, the different acoustic velocities between PDMS and the piezoelectric substrate will result in a change in the resonance frequency in the focal region. By considering these effects, an impedance boundary condition is applied to the PDMS walls. The impedance of PDMS can be expressed as $Z=\rho_{s}c_{s}$, where $\rho_{s}$ is the density of PDMS, and $c_{s}$ is the velocity at which the SAW propagates in PDMS.

**Figure 19:**
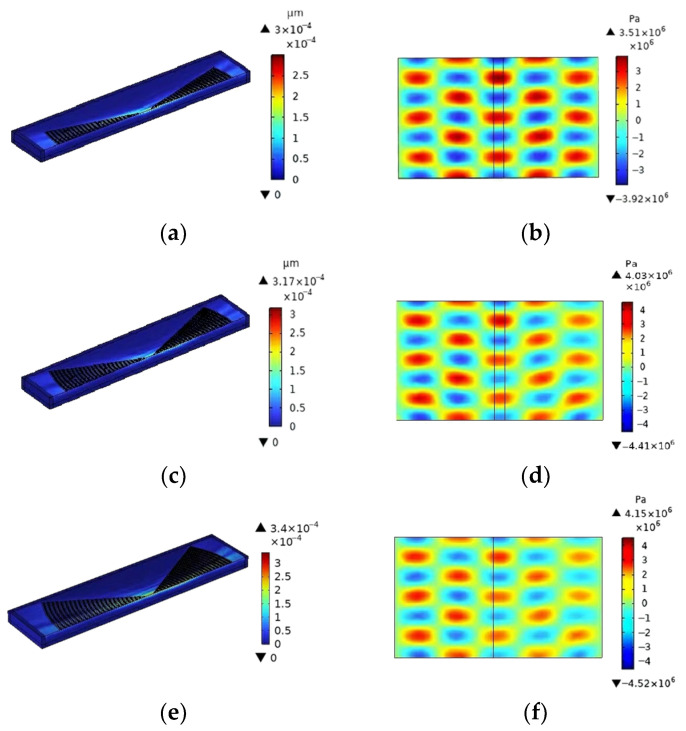
The effect of $D_{a}$ on SAW displacement and acoustic pressure. (**a**) SAW displacement for $D_{a}=30{}^{\circ}$ (Design 3); (**b**) acoustic pressure for $D_{a}=30{}^{\circ}$ (Design 3); (**c**) SAW displacement for $D_{a}=60{}^{\circ}$ (Design 4); (**d**) acoustic pressure for $D_{a}=60{}^{\circ}$ (Design 4); (**e**) SAW displacement for $D_{a}=90{}^{\circ}$ (Design 5); (**f**) acoustic pressure for $D_{a}=90{}^{\circ}$ (Design 5).

The effect of $D_{a}$ on the focusing properties of the EWC-FEFUDT is analyzed when $f_{L}=45\;µm$. The SAW total displacement and acoustic pressure distributions for different $D_{a}$ values are shown in Figure 19. The maximum displacement in Figure 19a,c,e is, respectively, $3\times 10^{-4}\;µm$, $3.17\times 10^{-4}\;µm,$ and $3.4\times 10^{-4}\;µm$. As the focusing angle increases, the focusing properties of the EWC-FEFUDT get better and better, which is similar to the FIDT [13]. The maximum acoustic pressure in Figure 19b,d,f is $3.51\times 10^{6}\;Pa$, $4.03\times 10^{-6}\;Pa$, and $4.15\times 10^{6}\;Pa$, respectively. Based on the above results, it is not difficult to find that the SAW energy of the EWC-FEFUDT in the focused region increases with an increasing $D_{a}$, both in the piezoelectric substrate and in the fluid domain; that is, the larger the $D_{a}$ of the EWC-FEFUDT and the larger the acoustic aperture of the EWC-FEFUDT, the better the focusing properties.

**Figure 20:**
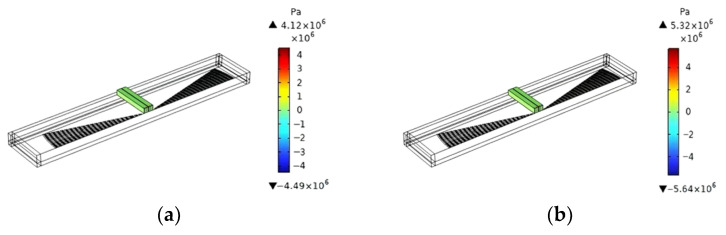

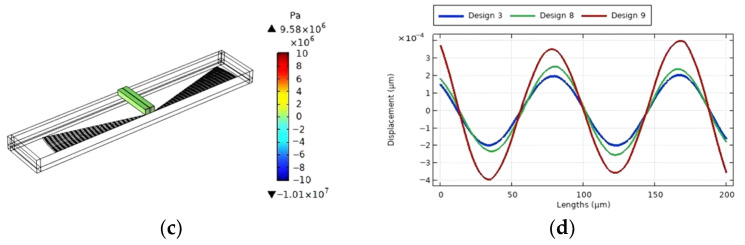
Effect of $f_{L}$ on acoustic pressure and SAW displacement. (**a**) $f_{L}=45\;µm$ (Design 3); (**b**) $f_{L}=65\;µm$ (Design 8); (**c**) $f_{L}=85\;µm$ (Design 9); (**d**) x component of SAW displacement in the Design 3 ($f_{L}=45\;µm$), Design 8 ($f_{L}=65\;µm$), and Design 9 ($f_{L}=85\;µm$).

When $D_{a}=30{}^{\circ}$, the effect of $f_{L}$ on the focusing properties of the EWC-FEFUDT is shown in Figure 20. As can be seen in Figure 20d, as the $f_{L}$ of the EWC-FEFUDT increases, the maximum value of the x component of SAW displacement increases; that is, the focusing properties of the SAW are improved. Normally, a longer $f_{L}$ will result in a larger propagation loss and a smaller acoustic pressure. However, in Figure 20a–c, the maximum value of acoustic pressure in the fluid region continuously increases with an increase in $f_{L}$. Because the longer the $f_{L}$ of the EWC-FEFUDT, the larger the acoustic aperture, the stronger the focusing properties, and the higher the acoustic pressure [38].

**Figure 21:**
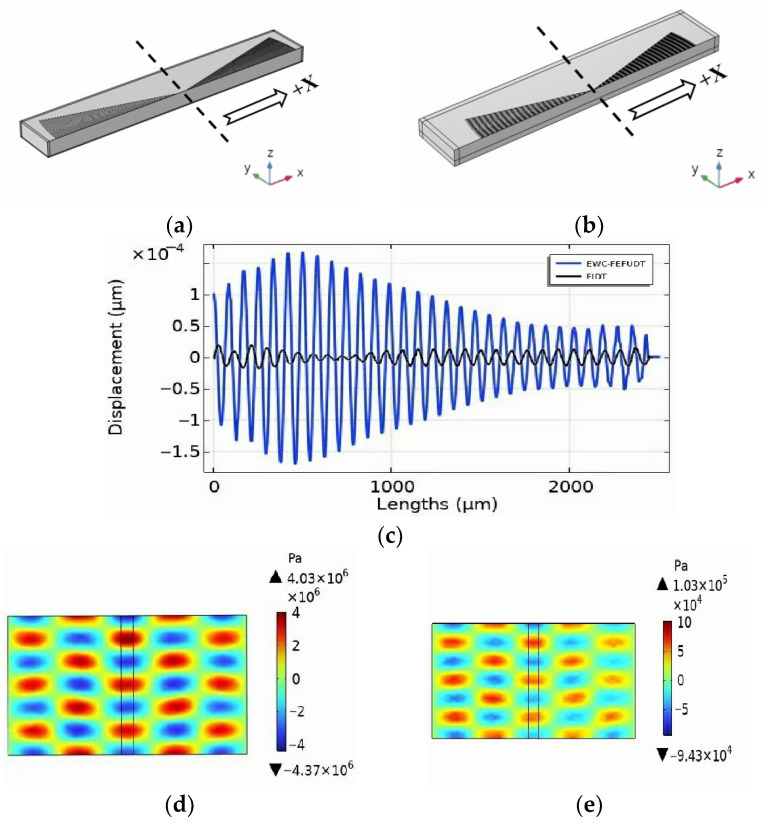
Substrate displacement and acoustic pressure distribution. (**a**) FEM model for calculating displacement amplitude in FIDT; (**b**) FEM model for calculating displacement amplitude in EWC-FEFUDT; (**c**) SAW displacements generated by FIDT and EWC-FEFUD in +X direction of (**a**,**b**); (**d**) acoustic pressure generated by EWC-FEFUDT; (**e**) acoustic pressure generated by FIDT.

The comparison of the focusing properties of the EWC-FEFUDT (Design 3) and the FIDT is shown in Figure 21. Figure 21a,b are models for simulating the SAW displacement distribution generated by the FIDT and EWC-FFUD, respectively. Figure 21c shows the SAW amplitudes generated by the EWC-FEFUDT and FIDT on the surface of the piezoelectric substrate along the x-direction in Figure 21a,b. Obviously, the SAW maximum amplitude of the EWC-FEFUDT is increased by about $1.5\times 10^{-4}\;µm$ compared to that of the FIDT. The acoustic pressures generated by the EWC-FEFUDT and FIDT in the fluid domain are shown in Figure 21d,e, respectively. The maximum acoustic pressure of the EWC-FEFUDT is an order of magnitude higher than that of the FIDT. The EWC-FEFUDT exhibits enhanced focusing properties.

### 3.5. Unidirectionality Analysis of EWC-FEFUDT

#### 3.5.1. Propagation Behaviors of SAW in EWC-FEFUDT

**Figure 22:**
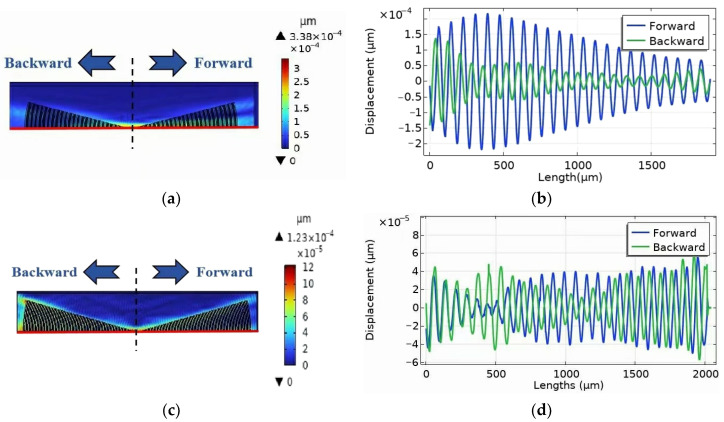
SAW displacements generated by EWC-FEFUDT and FIDT. (**a**) Total displacement generated by EWC-FEFUDT; (**b**) SAW displacement generated by EWC-FEFUDT along red line of (**a**); (**c**) Total displacement generated by FIDT; (**d**) SAW displacement generated by FIDT along red line of (**c**).

The unidirectionality of the EWC-FEFUDT can be given by the comparison of SAW amplitudes in the forward and backward directions. Figure 22b shows the SAW amplitude generated by the EWC-FEFUDT in both directions along the red line of Figure 22a. As can be seen, the SAW amplitude in the forward direction is significantly higher than that in backward direction, which proves the unidirectionality of the EWC-FEFUDT. Figure 22d shows the SAW amplitude generated by the FIDT in both directions along the red line of Figure 22c. In Figure 22d, no significant difference in the SAW amplitude in both directions generated by the FIDT is observed, which indicates the bidirectionality of the FIDT.

#### 3.5.2. Reflection Center and Transduction Center in EWC-FEFUDT

**Figure 23:**
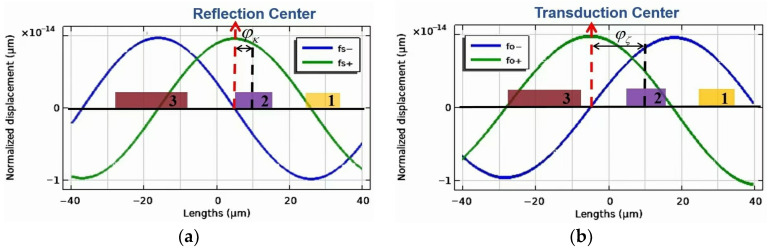
SAW standing wave distributions. (**a**) SAW standing waves under short-circuit conditions; (**b**) SAW standing waves under open circuit conditions.

According to the theory in Appendix A.3, when a transducer with its busbars is shorted, the reflection center can be calculated. In Figure 23, the normalized electrode thickness is 1%. Electrode 1 is connected to the ground. Electrode 2 is an active electrode. A voltage of 0 V is applied to the floating electrode 3, which is not involved in transduction.

The reflection center is a reference point relative to which the reflectivity of SAWs forward and backward is the same. Figure 23a shows the standing waves at the upper forbidden bandwidth edge ($f_{s+}$) and lower one ($f_{s-}$). As can be seen, both the nodes and crests of standing waves are displaced from the active electrode center by a phase angle $\phi_{\kappa }$ on the left. It means that for the EWC-FEFUDT, two standing waves are coupled to an active electrode at or near the lower band edge [14]. As a contrast, for the conventional bidirectional FIDT, only one standing wave is coupled. As a result, two acoustic standing waves are excited with nearly equal amplitudes at the center frequency.

The transduction center is a reference point relative to which both the amplitude and phase of the SAW generated forward and backward are the same. Figure 23b shows the standing waves at the upper forbidden bandwidth edge ($f_{o+}$) and the lower one ($f_{o-}$). Both the nodes and crests of standing waves are displaced from the active electrode center by a phase angle $\phi_{\zeta }$ on the left.

The displacements $x_{rc}$ and $x_{tc}$ of reflection and transduction centers from the active electrode center in the x-direction, respectively, are calculated as [41]:

(22) $$\left\{\begin{array}{c} x_{rc}=\frac{\lambda }{2\pi }\phi_{\kappa }\\ x_{tc}=\frac{\lambda }{2\pi }\phi_{\zeta } \end{array}\right.$$

According to Equation (22), $\phi_{\kappa }$ and $\phi_{\zeta }$ are calculated to be equal to −23° and −67.5°, respectively. We have published that the optimum value of the $\phi_{\kappa }-\phi_{\zeta }$ of the UDT is 45° [17]. In this paper, the direction angle of $\phi_{\kappa }-\phi_{\zeta }$ = 44.5° of the designed EWC-FEFUDT is obtained, being fairly close to the 45°.

#### 3.5.3. The Extraction of COM Parameters and the Analysis of Unidirectionality of the EWC-FEFUDT

**Figure 24:**
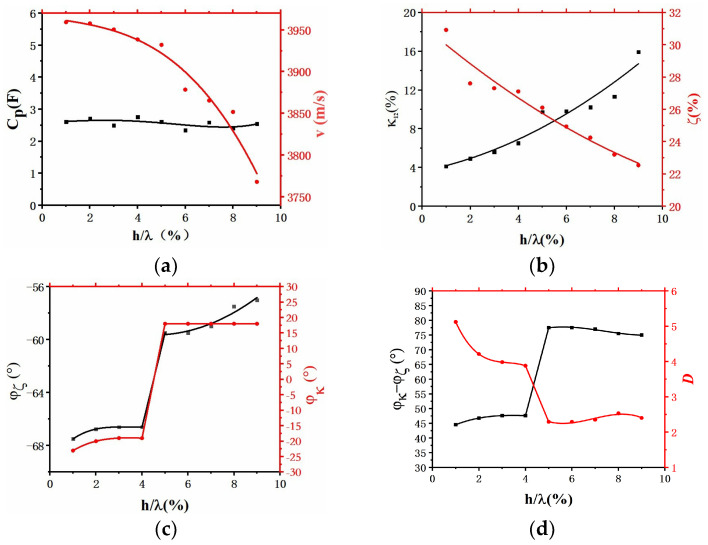
The relationships between COM parameters and normalized electrode thicknesses. (**a**) $C_{p}$ and v versus normalized electrode thickness; (**b**) $\kappa_{12}$ and ζ versus normalized electrode thickness; (**c**) $\phi_{\kappa }$ and $\phi_{\zeta }$ versus normalized electrode thickness; (**d**) $\phi_{\kappa }-\phi_{\zeta }$ and D versus normalized electrode thickness.

Based on the theories in Appendix A.3, Section 3 of the main text, and Section 3.5.2, $\kappa_{12}$, ζ, $C_{p}$, v, $\phi_{\kappa }$, and $\phi_{\zeta }$ can be obtained using the FEM. Figure 24 shows the relationships between these parameters and normalized electrode thicknesses. With an increasing electrode thickness, in Figure 24a, the $C_{p}$ does not change much but the v decreases due to the mass loading effect; in Figure 24b, the ζ decreases, but $\kappa_{12}$ increases continuously because of the dominant mechanical reflection relative to the electrical reflection; in Figure 24c, when the normalized electrode thickness (h/λ) is in the range of 1~4%, the $\phi_{\zeta }$ and $\phi_{\kappa }$ decrease slowly; when the h/λ is in the range of 4~5%, $\phi_{\zeta }$ decreases abruptly, while $\phi_{\kappa }$ switches from a negative value to a positive value; when the h/λ is in the range of 5~9%, the $\phi_{\zeta }$ decreases continuously, and $\phi_{\kappa }$ is almost unchanged.

The unidirectionality of the EWC-FEFUDT can be expressed by the directivity factor D [35]:

(23) $$D=\left|\frac{1+ie^{i2\left(\phi_{\kappa }-\phi_{\zeta }\right)}\tan h\left(\frac{N\left|\kappa_{12}\right|}{2}\right)}{1+ie^{-i2\left(\phi_{\kappa }-\phi_{\zeta }\right)}\tan h\left(\frac{N\left|\kappa_{12}\right|}{2}\right)}\right|$$

where N is the number of electrodes of the EWC-FEFUDT. When the directivity factor D > 1, it means that acoustic waves traveling backwards are stronger than those traveling forwards. When the directivity factor D < 1, the opposite is true. According to Equation (23), the relationship between D and the normalized electrode thickness is calculated for N = 72 and is shown in Figure 24d. When the h/λ is in the range of 1~4%, the directional angle ($\phi_{\kappa }-\phi_{\zeta }$) increases slowly, while the directivity factor (D) decreases; when the h/λ is in the range of 4~5%, $\phi_{\kappa }-\phi_{\zeta }$ and D, respectively, increase and decrease abruptly; when the h/λ is in the range of 5~9%, the changes in the $\phi_{\kappa }-\phi_{\zeta }$ and the D are small. When the electrode thickness is 1%, the directivity factor of the EWC-FEFUD is 5.12, and the directional angle of EWC-FEFUDT is 44.5°, which is the result in Section 3.5.2. When the normalized electrode thickness is approximately 1.2%, the directional angle of the EWC-FEFUDT is 45°, and the directionality of the proposed EWC-FEFUDT is optimal.

## 4. Discussion

Table 2 shows the structural parameters of the EWC-FEFUDT that influence the IL; that is, they have an impact on the energy utilization efficiency of SAW devices. Design 3 has low IL (−5.1 dB), and good unidirectionality (D = 5.12). Low IL indicates that less energy is lost during the conversion between electrical energy and mechanical energy in the EWC-FEFUDT, which is highly beneficial for the efficient operation of SAW devices. Table 3 compares the theoretical results of the IL and unidirectionality of the EWC-FEFUDT with other UDTs. The EWC-FEFUDT (Design 3) has reduced IL of 14.9 dB compared to the conventional FIDT [42]; the unidirectionality of the EWC-FEFUDT (Design 3) is 3.8 dB higher than that of the SPUDT [43]. The IL of the EWC-FEFUDT (Design 3) is increased by 2.6 dB compared to the EWC-FUDT [23], because the focusing angle of the EWC-FEFUDT is 30°, which is larger than that of the EWC-FUDT. This will lead to a stronger second-order effect such as SAW diffraction, refraction, and beam steering [13]. It can be seen from the comparison results that the EWC-FEFUDT (Design 3) tends to achieve a better balance between low IL and higher unidirectionality.

The enhancement of the acoustic pressure (by one order of magnitude) in the microchannel indicates that the proposed EWC-FEFUDT can generate stronger acoustic radiation forces, resulting in separating smaller particles. Jiang et al. [8] use two traditional IDTs with an operating frequency of 14.8 MHz (the SAW wavelength is 280 μm) to generate the SSAW field with a pressure amplitude of 0.45 MPa in the first separation area, which is enough to separate 25 μm and 5 μm particles. However, the drag force cancels most of the acoustic radiation force of the 15 μm and 5 μm particles, resulting in a small lateral displacement, so that the separation between 15 μm and 5 μm particles is not achieved. Although when 15 μm and 5 μm particles enter the second separation area, the 15 μm particles are separated by the high-frequency (19.98 MHz) ARF, the 5 μm particles still show little displacement. According to formula $F_{rad}=4\pi \phi \left(\tilde{\kappa },\tilde{\rho }\right)ka^{3}E_{ac}sin\left(2kx\right)$, the values of $F_{rad}$ of 15 μm and 5 μm particles in the second separation area are, respectively, $0.40325\pi^{2}\phi \left(\tilde{\kappa },\tilde{\rho }\right)$ and $0.014935\pi^{2}\phi \left(\tilde{\kappa },\tilde{\rho }\right)$. If the proposed EWC-FEFUDT is used, the corresponding values are, respectively, $72.517\pi^{2}\phi \left(\tilde{\kappa },\tilde{\rho }\right)$ and $2.6856\pi^{2}\phi \left(\tilde{\kappa },\tilde{\rho }\right)$ due to the improvement in the acoustic pressure (4.03 MPa). This indicates that 25 μm, 15 μm, and 5 μm particles can be simultaneously separated via single-stage separation. Furthermore, because the 5 μm particles can be displaced laterally, the particles with a size smaller than 5 μm can also be separated.

In a theoretical study, the surface roughness of the electrodes is ignored. In the actual application of the EWC-FEFUDT, the surface of the fabricated electrodes is not absolutely smooth, so the SAW will be scattered by the rough surface of the electrodes [44]. Then, the acoustic field distribution in the microchannel will vary because of the change in the direction of SAW propagation. As a result, the actual trajectories of the particles will deviate from the theoretical design value. So, in the future, the research will be focused on the experiments of separating particles using the proposed EWC-FEFUDT-based separation device.

## 5. Conclusions

In this paper, the performance of the EWC-FEFUDT is investigated using the COM theory. The COM parameter is extracted using the FEM, and the eigenmodes and dispersion characteristics of the EWC-FEFUDT are obtained. The four different forbidden band edge frequencies account for the unidirectionality of the proposed EWC-FEFUDT. The phase shifts of the reflection center and transduction center in the EWC-FEFUDT are determined from the standing SAW. The direction angle of $\phi_{\kappa }-\phi_{\zeta }$ = 44.5° of the EWC-FEFUDT (Design 3) is obtained, being fairly close to the optimum value of 45°. The EWC-FEFUDT (Design 3) has a lower IL of −5.1 dB and greater unidirectionality (D = 5.12). The SAW maximum amplitude of the EWC-FEFUDT (Design 3) is increased by about $1.5\times 10^{-4}\;µm$ compared to that of the FIDT. The maximum acoustic pressure of the EWC-FEFUDT is an order of magnitude higher than that of the FIDT. The EWC-FEFUDT exhibits enhanced focusing properties. The proposed EWC-FEFUDT has great prospects in separating particles in the fluid domain.

## Figures and Tables

**Table 1:** Boundary conditions used in the simulations.

Boundary	Electrical Boundary Conditions	Mechanical Boundary Conditions
$\Gamma_{1}$	Ground	Fixed
$\Gamma_{2}$	Continuity	Free
$\Gamma_{R},\Gamma_{L}$	Periodic	Periodic
Electrode (3)	∫−n·J=0	/
Electrode (2)	1 V	/
Electrode (1)	Ground	/

**Table 2:** Designed structural parameters of the EWC-FEFUDT.

Design	Number of Electrode Pairs (*n*)	Focusing Angle $D_{a}$ (deg)	Focus Distance *f_L_* (μm)	Delay Distance *L* (μm)	Electrode Thickness *h* (μm)
Design 1	8	30	45	130	0.8
Design 2	16	30	45	130	0.8
Design 3	24	30	45	130	0.8
Design 4	24	60	45	130	0.8
Design 5	24	90	45	130	0.8
Design 6	24	30	45	90	0.8
Design 7	24	30	45	170	0.8
Design 8	24	30	65	130	0.8
Design 9	24	30	85	130	0.8
Design 10	24	30	45	130	0.6
Design 11	24	30	45	130	1.0

**Table 3:** The comparison of IDT performances.

Paper	IDT	Centre Frequency	$D_{a}$	n	IL	$20\times \log_{10}(D)$
This paper	EWC-FEFUDT (Design 3)	50 MHz	30°	24	−5.1 dB	13.8 dB
[23]	EWC-FUDT	1 GHz	5°	20	−2.5 dB	/
[43]	SPUDT	350 MHz	/	20	/	10 dB
[42]	FIDT	60 MHz	20°	10	−20 dB	/

**Figure A1:**
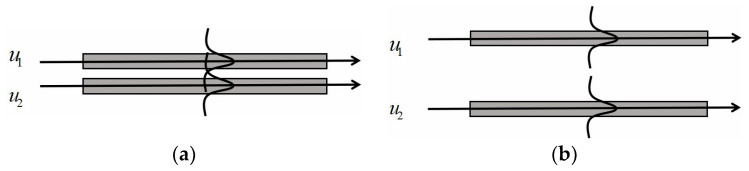
Two fibers. (**a**) Coupled fibers; (**b**) fibers without coupling.

**Figure A2:**
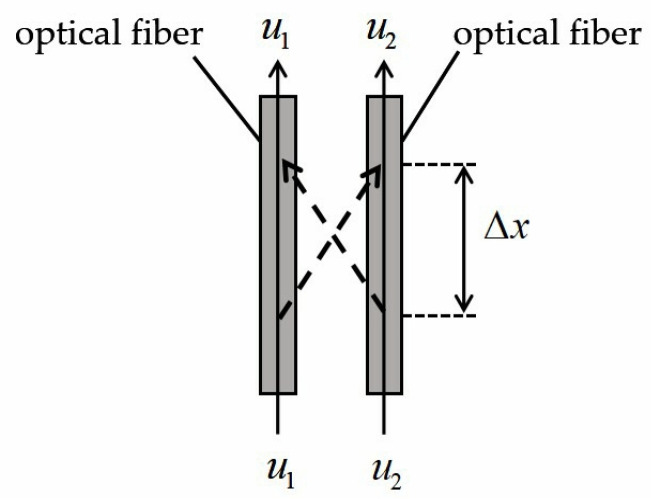
Input and output for an arbitrary section.

**Figure A3:**
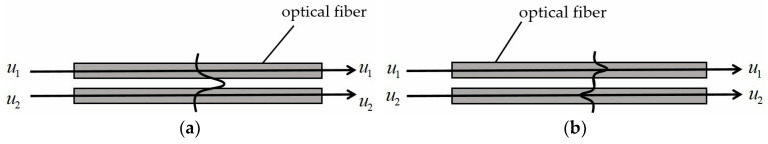
Symmetric and anti-symmetric modes. (**a**) Symmetric modes; (**b**) anti-symmetric modes.

**Figure A4:**
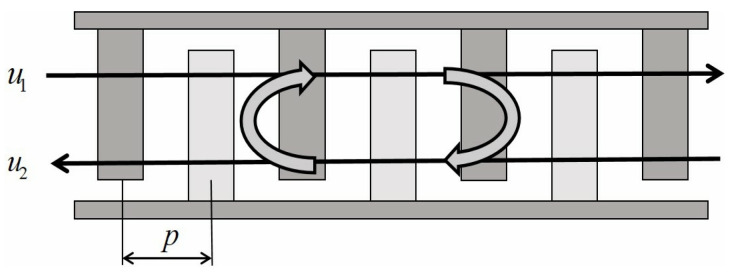
Periodic structure.

**Figure A5:**
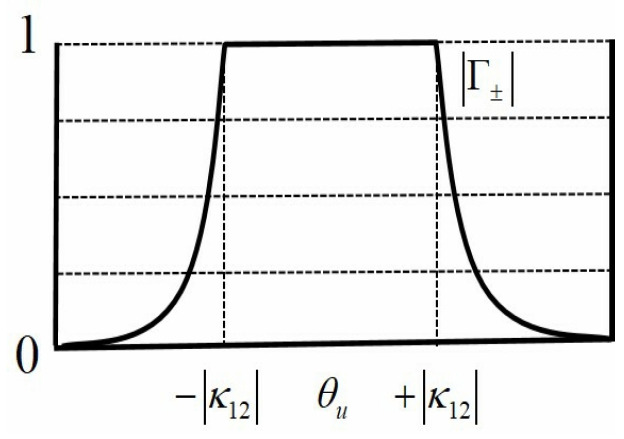
Reflection coefficient $\Gamma_{\pm }$ of periodic structure.

**Figure A6:**
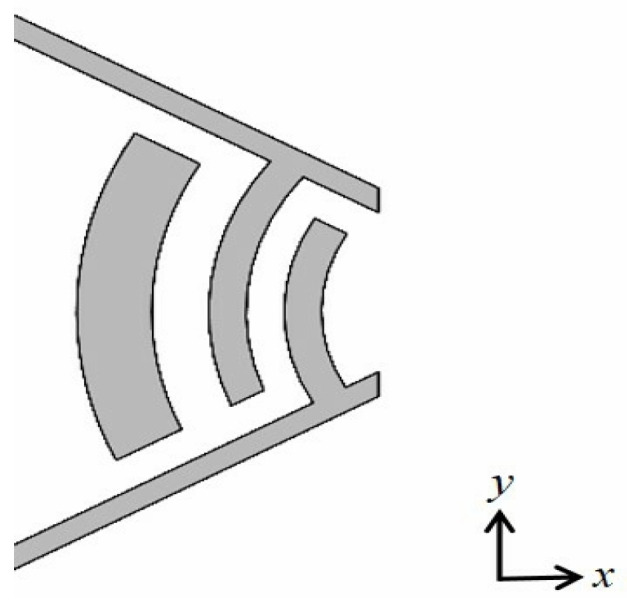
Single-periodic structure of the EWC-FEFUDT.

**Figure A7:**
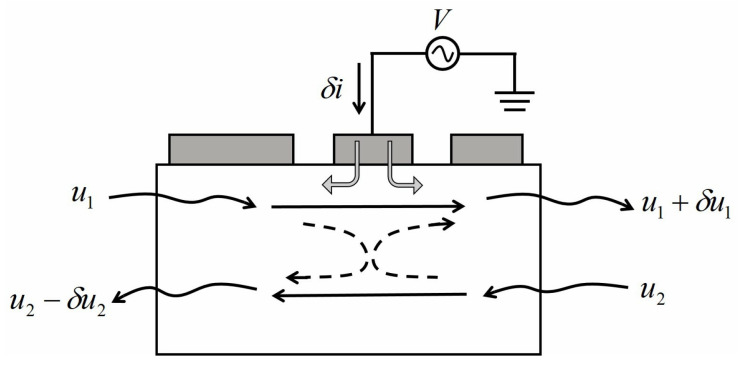
Unit section of EWC-FEFUDT.

**Figure A8:**
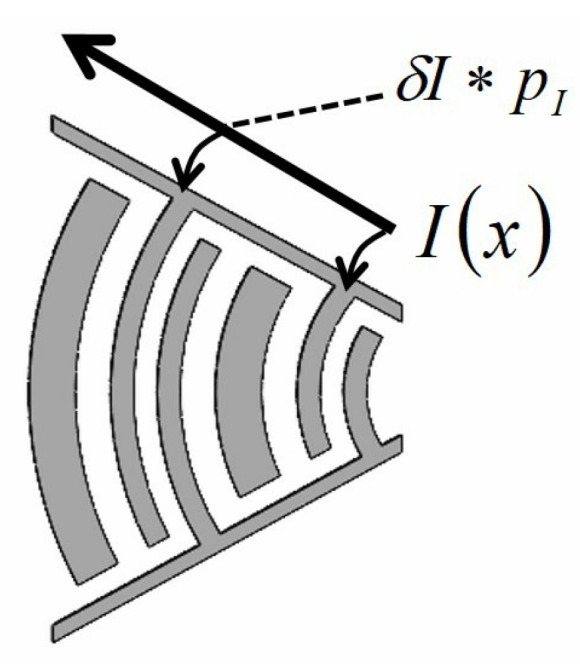
Current on busbar of EWC-FEFUDT.

**Figure A9:**
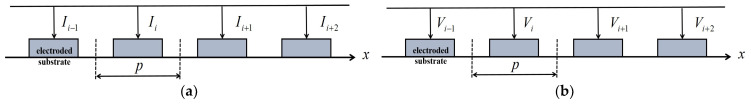
(**a**) Shorted-circuited gratings; (**b**) open-circuited gratings.

## Data Availability

The original contributions presented in the study are included in the article, and further inquiries can be directed to the corresponding author.

## Appendix A. Derivation of the COM Equations Using the Perturbation Theory

### Appendix A.1. The Derivation of the COM Equations

Eigenmodes are waves propagating without changing their amplitude, and they do not exchange their energy among themselves. Hence, the amplitude $u_{n}\left(x\right)$ of the *n*-th eigenmode propagating in the +x direction satisfies the following differential equation [31]:

(A1) $$\frac{\partial u_{n}\left(x\right)}{\partial x}=-j\beta u_{n}\left(x\right)$$

where β is the wavenumber of the mode. The mode amplitude is implicitly assumed to be normalized so that modes with ${\left|u_{n}\right|}^{2}=1$ carry unit power.

The mutual coupling of eigenmodes $u_{1}$ and $u_{2}$ can be considered as a case of two optical fibers placed in close proximity to each other, as shown in Figure A1a. The wave coupling and, thus, the transfer of optical power between the fibers can occur due to the interaction of the extended evanescent optical fields outside of the cores. On the contrary, mutual coupling of modes cannot occur when two optical fibers are located farther apart, as shown in Figure A1b. If the coupling between the two eigenmodes is small, its contribution may be expressed by adding linear perturbation terms in Equation (A1):

(A2) $$\frac{\partial u_{1}\left(x\right)}{\partial x}=-j\beta u_{1}\left(x\right)-j\kappa u_{2}\left(x\right)$$

(A3) $$\frac{\partial u_{2}\left(x\right)}{\partial x}=-j\beta u_{2}\left(x\right)-j\kappa^{\prime }u_{1}\left(x\right)$$

These types of simultaneous differential equations with linear mutual coupling terms are called COM equations, in which κ and $\kappa^{\prime }$ are the parameters responsible for the mutual coupling and are called mutual coupling coefficients.

The mode-coupled system is assumed to be unitary, i.e., without any losses. Since the mode amplitudes are normalized, the unitary condition for the section shown in Figure A2 is given by:

(A4) $$\frac{{\left|u_{1}\left(x+\Delta x\right)\right|}^{2}+{\left|u_{2}\left(x+\Delta x\right)\right|}^{2}-{\left|u_{1}\left(x\right)\right|}^{2}-{\left|u_{2}\left(x\right)\right|}^{2}}{\Delta x}=0$$

Since this condition must be fulfilled for arbitrary x and Δx, consequently, setting Δx→0 gives:

(A5) $$\frac{\partial \left({\left|u_{1}\left(x\right)\right|}^{2}+{\left|u_{2}\left(x\right)\right|}^{2}\right)}{\partial x}=0$$

The substitution of Equations (A2) and (A3) into Equation (A5) gives:

(A6) $$u_{1}\frac{\partial u_{1}{\left(x\right)}^{*}}{\partial x}+{u_{1}}^{*}\frac{\partial u_{1}\left(x\right)}{\partial x}+u_{2}\frac{\partial u_{2}{\left(x\right)}^{*}}{\partial x}+{u_{2}}^{*}\frac{\partial u_{2}\left(x\right)}{\partial x}=\;2\varsigma \left(\beta \right)\left({\left|u_{1}\left(x\right)\right|}^{2}+{\left|u_{2}\left(x\right)\right|}^{2}\right)+2\varsigma \left\{\left(\kappa -\kappa^{\prime *}\right)u_{1}{\left(x\right)}^{*}u_{2}\left(x\right)\right\}=0$$

where ∗ denotes the conjugate. So that the condition of Equation (A6) holds for arbitrary $u_{n}$, the relations $\varsigma \left(\beta \right)=0$ and $\kappa =\kappa^{\prime *}\left(\kappa^{\prime }=\kappa^{*}\right)$ must be satisfied. Then, the substitution of these relations into Equations (A2) and (A3) gives the final COM equations, i.e.,

(A7) $$\frac{\partial u_{1}\left(x\right)}{\partial x}=-j\beta u_{1}\left(x\right)-j\kappa u_{2}\left(x\right)$$

(A8)
$$\frac{\partial u_{2}\left(x\right)}{\partial x}=-j\kappa^{*}u_{1}\left(x\right)-j\beta u_{2}\left(x\right)$$

If the two fibers are equivalent to each other, the subscripts 1 and 2 are exchangeable. Hence, from Equations (A7) and (A8), we get $\kappa =\kappa^{*}$.

Defining differential operator ∂/∂x≡Λ, Equations (A7) and (A8) can be written in matrix form:

(A9) $$\left(\begin{array}{cc} \Lambda +j\beta & j\kappa \\ j\kappa^{*} & \Lambda +j\beta \end{array}\right)\left(\begin{array}{c} u_{1}\left(x\right)\\ u_{2}\left(x\right) \end{array}\right)=\left(\begin{array}{c} 0\\ 0 \end{array}\right)$$

For the existence of nontrivial solutions, the determinant of the matrix on the left-hand side must be zero. Then, we get:

(A10) $${\left(\Lambda +j\beta \right)}^{2}+{\left|\kappa \right|}^{2}=0$$

and its solutions are given by

(A11) $$\Lambda =-j\beta \mp j\left|\kappa \right|\equiv -j\theta_{\pm }$$

The solutions of Equation (A9) can be written as a linear combination of solutions of the following linear differential equation.

(A12) $$\Lambda u_{i}=\frac{\partial u_{i}}{\partial x}=-j\theta_{\pm }u_{i}$$

Thus, the solutions of Equations (A7) and (A8) can be obtained as:

(A13) $$u_{1}\left(x\right)=c_{1}\exp\left(-j\theta_{+}x\right)+c_{2}\exp\left(-j\theta_{-}x\right)$$

(A14) $$u_{2}\left(x\right)=r_{+}c_{1}\exp\left(-j\theta_{+}x\right)+r_{-}c_{2}\exp\left(-j\theta_{-}x\right)$$

where $c_{1}$ and $c_{2}$ is a constant determined by the boundary condition, and $r_{\pm }=\left[\theta_{\pm }-\beta \right]/\kappa =\pm \left|\kappa \right|/\kappa$. As is clear from Equations (A13) and (A14), $\theta_{\pm },$ defined in Equation (A11), corresponds to the wavenumbers of two modes when coupling occurs. If the two fibers are equivalent to each other, $r_{\pm }=1$ because $\kappa =\kappa^{*}$. Then, it is clear that $u_{1}\left(x\right)=u_{2}\left(x\right)$ for $\theta_{+},$ whereas $u_{1}\left(x\right)=-u_{2}\left(x\right)$ for $\theta_{-}$. Namely, the former and latter correspond to symmetric and anti-symmetric modes, respectively, as shown in Figure A3.

### Appendix A.2. The COM Equations of Periodic Structures

In SAW devices, the eigenmodes always propagate in a periodic interdigital array. Therefore, it is necessary to consider the coupling of two modes propagating in the periodic structure shown in Figure A4 with periodicity p, where one mode $u_{1}$ (mode amplitude) propagates in the +x direction and the other mode $u_{2}$ (mode amplitude) propagates in the −x direction.

Assuming this periodic structure is of infinite length and each period is equivalent to every other, then the field distribution $u_{n}\left(x\right)$ of the *n*-th eigenmode in each period is the same as that in every other. That is, $u_{n}\left(x\right)$ satisfies the Floquet theory [31]:

(A15) $$u_{n}\left(x+p\right)=u_{n}\left(x\right)\exp\left(-j\beta p\right)$$

which defines the function $U\left(x\right)$:

(A16) $$U\left(x\right)=u_{n}\left(x\right)\exp\left(-j\beta p\right)$$

Floquet theory indicates that $U\left(x\right)$ must be a periodic function with periodicity p. So, $U\left(x\right)$ can be expressed in terms of the Fourier expansion.

(A17) $$U\left(x\right)=\sum_{n=-\infty }^{+\infty }A_{n}\exp\left(-2\pi jnx/p\right)$$

Combining Equations (A16) and (A17) yields:

(A18) $$u_{n}\left(x\right)=\sum_{n=-\infty }^{+\infty }A_{n}\exp\left\{-j\left(\beta +2n\pi /p\right)x\right\}$$

where $A_{n}$ is the eigenmode’s amplitude. This relation suggests that the field in the periodic structure with periodicity p is expressed as a sum of sinusoidal waves with discrete wavenumbers $\beta_{n}=2n\pi /p+\beta$. In other words, wave components with spatial frequency 2nπ/p+β are generated by the spatial modulation of the incident wave with β. Wave fields scattered at each period may interfere. Usually, over wide frequency bands, they cancel each other, and the total reflected field will become negligible. In this case, the wave can be accurately described in Equation (A18) by a single term with n=0. However, in a certain frequency band, the scattered waves are in phase, and they will sum up and cause very strong reflection. In this case, multiple terms in Equation (A18) must be used for an accurate description of the wave field. This situation is called Bragg reflection.

According to Equation (A18), the *n*-th order Bragg reflection occurs at $\left|\beta -2\pi n/p\right|≅\left|\beta \right|$. This means that the most significant coupling is what between the component with wavenumber β and that with wavenumber β−2πn/p, and contributions by the others are relatively small. Thus, by taking only these two components into account, we obtain the COM equations:

(A19) $$\frac{\partial u_{1}\left(x\right)}{\partial x}=-j\beta u_{1}\left(x\right)-j\kappa_{12}u_{2}\left(x\right)\exp\left(-2\pi jnx/p\right)$$

(A20) $$\frac{\partial u_{2}\left(x\right)}{\partial x}=+j\beta u_{2}\left(x\right)+j\kappa_{12}^{\prime }u_{1}\left(x\right)\exp\left(+2\pi jnx/p\right)$$

where $\kappa_{12}$ and $\kappa_{12}^{\prime }$ are the mutual coupling coefficients. By using the following expressions:

(A21) $$u_{1}\left(x\right)=U_{1}\left(x\right)\exp\left\{-\pi jnx/p\right\}$$

(A22) $$u_{2}\left(x\right)=U_{2}\left(x\right)\exp\left\{+\pi jnx/p\right\}$$

Equations (A19) and (A20) can be rewritten as:

(A23) $$\frac{\partial U_{1}\left(x\right)}{\partial x}=-j\theta_{u}U_{1}\left(x\right)-j\kappa_{12}U_{2}\left(x\right)$$

(A24) $$\frac{\partial U_{2}\left(x\right)}{\partial x}=+j\kappa_{12}^{\prime }U_{1}\left(x\right)+j\theta_{u}U_{2}\left(x\right)$$

where $\theta_{u}=\beta -\pi n/p$ is the deviation of the wavenumber β from the Bragg condition and is called the detuning factor. To derive the unitary condition, the same procedure in Appendix A.1 is followed:

(A25)
$$\begin{array}{l} \frac{\partial \left({\left|U_{1}\left(x\right)\right|}^{2}-{\left|U_{2}\left(x\right)\right|}^{2}\right)}{\partial x}=\\ U_{1}\frac{\partial U_{1}{\left(x\right)}^{*}}{\partial x}+{U_{1}}^{*}\frac{\partial U_{1}\left(x\right)}{\partial x}-U_{2}\frac{\partial U_{2}{\left(x\right)}^{*}}{\partial x}-{U_{2}}^{*}\frac{\partial U_{2}\left(x\right)}{\partial x}=\\ 2\varsigma \left(\theta_{u}\right)\left({\left|U_{1}\left(x\right)\right|}^{2}+{\left|U_{2}\left(x\right)\right|}^{2}\right)+2\varsigma \left\{\left(\kappa_{12}-\kappa_{12}^{\prime *}\right)U_{1}{\left(x\right)}^{*}U_{2}\left(x\right)\right\}=0 \end{array}$$

The unitary condition of $\varsigma \left(\theta_{u}\right)=0$ and $\kappa_{12}=\kappa_{12}^{\prime *}\left(\kappa_{12}^{\prime }=\kappa_{12}^{*}\right)$ is obtained. Thus, the final COM equations is given by:

(A26) $$\frac{\partial U_{1}\left(x\right)}{\partial x}=-j\theta_{u}U_{1}\left(x\right)-j\kappa_{12}U_{2}\left(x\right)$$

(A27) $$\frac{\partial U_{2}\left(x\right)}{\partial x}=+j\kappa_{12}^{*}U_{1}\left(x\right)+j\theta_{u}U_{2}\left(x\right)$$

If the structure is symmetric, we get $\kappa_{12}=\kappa_{12}^{*}$. By employing the same procedure in Appendix A.1, Equations (A26) and (A27) can also be written in matrix form by defining the differential operator ∂/∂x≡Λ. For the existence of nontrivial solutions, the determinant of the matrix must be zero; that is:

(A28) $$\Lambda =\mp j\sqrt{\theta_{u}-{\left|\kappa_{12}\right|}^{2}}\equiv \mp j\theta_{p}$$

By using this, the solution can be expressed in the form:

(A29) $$U_{1}\left(x\right)=c_{1}\exp\left(-j\theta_{P}x\right)+\Gamma_{-}c_{2}\exp\left(+j\theta_{P}x\right)$$

(A30) $$U_{2}\left(x\right)=\Gamma_{+}c_{1}\exp\left(-j\theta_{P}x\right)+c_{2}\exp\left(+j\theta_{P}x\right)$$

where $\Gamma_{+}=\left(\theta_{p}-\theta_{u}\right)/\kappa_{12}$ and $\Gamma_{-}=\left(\theta_{p}-\theta_{u}\right)/\kappa_{12}^{*}$, which, respectively, correspond to the reflection coefficient for SAW incidence on a semi-infinite grating from the ∓x direction. $\theta_{p}$ is the wavenumber of the perturbed mode coupled with the reflected wave. The reflection coefficient takes a maximum at $\theta_{u}=0$.

Figure A5 shows $\Gamma_{\pm }$ as a function of frequency. When $\left|\Gamma_{\pm }\right|=1$, the corresponding frequency range is called the forbidden band, the width of which is determined by $\left|\kappa_{12}\right|$.

### Appendix A.3. Analysis of EWC-FEFUDT Based on COM Theory

Figure A6 shows a single-periodic structure of the EWC-FEFUDT in the Cartesian coordinate system, where the widths of the electrodes are unequal in the x-direction.

Figure A7 shows a unit section the EWC-FEFUDT, where V is the voltage applied. Following the general derivation procedure of the COM equations described in Appendix A.2, based on Equations (A19) and (A20), the COM equations for the EWC-FEFUDT when the voltage V is applied can be written as:

(A31) $$\frac{\partial u_{1}\left(x\right)}{\partial x}=-j\beta u_{1}\left(x\right)-j\kappa_{12}u_{2}\left(x\right)\exp\left[\left(-2\pi jnx/p\right)\right]+j\zeta V\exp\left[\left(-\pi jnx/p\right)\right]$$

(A32) $$\frac{\partial u_{2}\left(x\right)}{\partial x}=+j\beta u_{2}\left(x\right)+j\kappa_{12}^{\prime }u_{1}\left(x\right)\exp\left[\left(+2\pi jnx/p\right)\right]-j\zeta^{\prime }V\exp\left[\left(+\pi jnx/p\right)\right]$$

where ζ is the coefficient responsible for the excitation efficiency and is called the transduction coefficient. When the propagation constants of $u_{1}$ and $u_{2}$ deviate from the Bragg condition, they can be expressed as:

(A33) $$u_{1}\left(x\right)=U_{1}\left(x\right)\exp\left\{-j\left(\frac{n\pi }{p}+\theta_{u}\right)x\right\}$$

(A34) $$u_{2}\left(x\right)=U_{2}\left(x\right)\exp\left\{+j\left(\frac{n\pi }{p}+\theta_{u}\right)x\right\}$$

Substituting Equations (A33) and (A34) into the Equations (A31) and (A32) yields [45,46].

(A35) $$\frac{\partial U_{1}\left(x\right)}{\partial x}=-j\kappa_{11}U_{1}\left(x\right)-j\kappa_{12}U_{2}\left(x\right)\exp\left(j2\theta_{u}x\right)+j\zeta V\exp\left(j\theta_{u}x\right)$$

(A36) $$\frac{\partial U_{2}\left(x\right)}{\partial x}=j\kappa_{11}U_{2}\left(x\right)+j\kappa_{12}^{\prime }\exp\left(-j2\theta_{u}x\right)U_{1}\left(x\right)-j\zeta^{\prime }V\exp\left(-j\theta_{u}x\right)$$

where $\kappa_{11}$ is called the self-coupling coefficient.

As shown in Figure A8, the increment of the current $I\left(x\right)$ within a period $p_{I}$ of the EWC-FEFUDT is given by:

(A37) $$I\left(x+p_{I}\right)-I\left(x\right)=p_{I}\left[-j\eta \exp\left(-j\theta_{u}x\right)U_{1}\left(x\right)-j\eta^{\prime }\exp\left(j\theta_{u}x\right)U_{2}\left(x\right)+j\omega CV\right]$$

where C is the static capacitance per unit length of the electrode, and η and $\eta^{\prime }$ are the coefficients responsible for the receiving efficiency. By taking the limit with respect to $p_{I}$, we obtain:

(A38) $$\frac{\partial I\left(x\right)}{\partial x}=-j\eta \exp\left(-j\theta_{u}x\right)U_{1}\left(x\right)-j\eta^{\prime }\exp\left(j\theta_{u}x\right)U_{2}\left(x\right)+j\omega CV$$

The unitary conditions for the EWC-FEFUDT can be obtained according to Equations (A35) to (A38). Although $u_{1}$ and $u_{2}$ are effective values, we express V and $I\left(x\right)$ at their peak amplitudes. Then, from the power conservation relation:

(A39) $$\frac{\partial \left({\left|u_{1}\left(x\right)\right|}^{2}-{\left|u_{2}\left(x\right)\right|}^{2}\right)}{\partial x}=\frac{1}{2}\frac{\partial R\left[VI{\left(x\right)}^{*}\right]}{\partial x}$$

The relations $\varsigma \left(\beta \right)=0$, $\kappa_{12}^{\prime }=\kappa_{12}^{*}$, η=4ζ, and $\eta^{\prime }=4\zeta^{\prime }$ are obtained. Added to this, in the case where the EWC-FEFUDT is driven by a single-phase electrical source, the relation $\zeta^{\prime }=\zeta^{*}$ holds. Therefore, the Equation (A38) can be rewritten as (A40):

(A40) $$\frac{\partial I\left(x\right)}{\partial x}=-4j\zeta \exp\left(-j\theta_{u}x\right)U_{1}\left(x\right)-4j\zeta^{*}\exp\left(j\theta_{u}x\right)U_{2}\left(x\right)+j\omega CV$$

Equations (A35), (A36), and (A40) are the final COM equations for the EWC-FEFUDT. From the COM parameters, the detuning coefficient $\theta_{u}$ can be rewritten as Equation (A41):

(A41) $$\theta_{u}=\frac{2\pi }{\lambda }\left(\frac{f}{f_{0}}-1\right)$$

where f is the frequency of the eigenmodes, and $f_{0}$ is the Bragg frequency.

In a conventional loss-less symmetric IDT, $\kappa_{12}$ and ζ are real, while these are complex in an asymmetric IDT such as the EWC-FEFUDT. So, we define $\kappa_{12}$ and ζ as follows [17]:

(A42) $$\kappa_{12}=\left|\kappa_{12}\right|\exp\left(j2\phi_{\kappa }\right)$$

(A43) $$\zeta =\left|\zeta \right|\exp\left(j\phi_{\zeta }\right)$$

where $2\phi_{\kappa }$ and $\phi_{\zeta }$ are, respectively, the phases of reflection and transduction.

The extraction process of COM parameters requires the consideration of the electrode structure in both short-circuited and open-circuited cases.

For a short-circuited grating, as shown in Figure A9a, a zero voltage is applied to the terminal, and the COM Equations (A35) and (A36) for the EWC-FEFUDT can be simplified to:

(A44) $$\frac{\partial U_{1}\left(x\right)}{\partial x}=-j\kappa_{11}U_{1}\left(x\right)-j\kappa_{12}\exp\left(j2\theta_{u}x\right)U_{2}\left(x\right)$$

(A45) $$\frac{\partial U_{2}\left(x\right)}{\partial x}=j\kappa_{11}U_{2}\left(x\right)+j\kappa_{12}^{*}\exp\left(-j2\theta_{u}x\right)U_{1}\left(x\right)$$

Employing the same procedure in Appendix A.2, Equations (A44) and (A45) can also be written in matrix form by defining the differential operator ∂/∂x≡Λ. For the existence of nontrivial solutions, the determinant of the matrix must be zero; that is:

(A46) $$\Lambda =\mp j\sqrt{{\left(\theta_{u}+\kappa_{11}\right)}^{2}-{\left|\kappa_{12}\right|}^{2}}\equiv \mp j\theta_{p}$$

Let $\theta_{S}=\theta_{u}+\kappa_{11}$; the SAW dispersion equation is:

(A47) $$\theta_{p}=\sqrt{{\left(\theta_{S}\right)}^{2}-{\left|\kappa_{12}\right|}^{2}}$$

The frequency region with the pronounced attenuation, $\theta_{S}\leq \left|\kappa_{12}\right|$, is denoted as the forbidden band. The lower and upper frequencies $f_{s-}$ and $f_{s+}$ of the forbidden band can be obtained by considering the zero of the dispersion relation as, i.e., make $\theta_{p}=0$.

(A48) $$f_{s+}=\left(1+\frac{\left|\kappa_{12}\right|\lambda }{2\pi }-\frac{\kappa_{11}\lambda }{2\pi }\right)f_{0}$$

(A49)
$$f_{s-}=\left(1-\frac{\left|\kappa_{12}\right|\lambda }{2\pi }-\frac{\kappa_{11}\lambda }{2\pi }\right)f_{0}$$

The width of the forbidden band is proportional to the mutual coupling coefficients $\kappa_{12}$. According to the Equations (A48) and (A49), the center frequency $f_{0}$ can be obtained:

(A50) $$f_{0}=\frac{f_{s+}+f_{s-}}{2}$$

For an open-circuited grating, as shown in Figure A9b, the electrode voltages are floating, and the current density ∂I/∂x is equal to zero. We can obtain the following equation:

(A51) $$V=\frac{2\zeta^{*}}{\omega C}\exp\left(-j\theta_{u}x\right)U_{1}\left(x\right)+\frac{2\zeta }{\omega C}\exp\left(j\theta_{u}x\right)U_{2}\left(x\right)$$

Substituting Equation (A51) into Equations (A35) and (A36), we have:

(A52) $$\frac{\partial U_{1}\left(x\right)}{\partial x}=-j\left[\kappa_{11}-\frac{2{\left|\zeta \right|}^{2}}{\omega C}\right]U_{1}\left(x\right)-j\kappa_{b}\exp\left(j2\theta_{u}x\right)U_{2}\left(x\right)$$

(A53) $$\frac{\partial U_{2}\left(x\right)}{\partial x}=j\left[\kappa_{11}-\frac{2{\left|\zeta \right|}^{2}}{\omega C}\right]U_{2}\left(x\right)+j\kappa_{b}^{*}\exp\left(-j2\theta_{u}x\right)U_{1}\left(x\right)$$

Equations (A52) and (A53) are expressed in the same form as (A44) and (A45), meaning that they have similar solutions. However, the mutual coupling coefficient is $\kappa_{b}=\kappa_{12}-2\zeta^{2}/\omega C$. The SAW dispersion equation is:

(A54) $$\theta_{p}^{\prime }=\sqrt{{\left|\kappa_{12}\right|}^{2}-2\left|\kappa_{12}\right|\frac{2{\left|\zeta \right|}^{2}}{\omega C}\cos2\left(\phi_{\kappa }-\phi_{\zeta }\right)+{\left(\frac{2{\left|\zeta \right|}^{2}}{\omega C}\right)}^{2}-{\left(\theta_{u}+\kappa_{11}-\frac{2{\left|\zeta \right|}^{2}}{\omega C}\right)}^{2}}$$

At the forbidden band edges of the open-circuited grating, we have $\theta_{p}^{\prime }=0$. The corresponding upper and lower frequencies $f_{o+}$ and $f_{o-}$ are as follows:

(A55) $$f_{o+}=\left(1+\frac{\lambda }{2\pi }\frac{2{\left|\zeta \right|}^{2}}{\omega C}+\frac{\lambda }{2\pi }\left|\kappa_{b}\right|-\frac{\kappa_{11}\lambda }{2\pi }\right)f_{0}$$

(A56) $$f_{o-}=\left(1+\frac{\lambda }{2\pi }\frac{2{\left|\zeta \right|}^{2}}{\omega C}-\frac{\lambda }{2\pi }\left|\kappa_{b}\right|-\frac{\kappa_{11}\lambda }{2\pi }\right)f_{0}$$
